# Phenylboronic Acid‐Modified Copper Nanozymes as Nanoscavengers of Bacterial Polysaccharides Causing Acute Lung Injury

**DOI:** 10.1002/advs.202513536

**Published:** 2025-09-16

**Authors:** Haojie Ge, Min Wang, Haijiao Xie, Xu‐Lin Chen, Xianwen Wang

**Affiliations:** ^1^ Department of Burns The First Hospital Affiliated of Anhui Medical University Anhui Medical University Hefei Anhui 230032 P. R. China; ^2^ School of Biomedical Engineering Anhui Medical University Hefei 230032 P. R. China; ^3^ Hangzhou Yanqu Information Technology Co., Ltd Hangzhou 310003 P. R. China

**Keywords:** acute lung injury, copper nanoparticles, nanozymes, polysaccharides, target bacteria

## Abstract

Acute lung injury (ALI), especially burn‐induced cases complicated by secondary infections and hyperinflammation, remains challenging to treat. This study developed 4‐mercaptobenzoic acid (MPBA)‐modified copper nanozymes (CuMPBA) to simultaneously combat bacterial infections and toxin‐triggered immune overactivation. CuMPBA binds bacterial surface polysaccharides via boronate ester bonds, neutralizing lipopolysaccharides (LPS) and lipoteichoic acid (LTA). It demonstrates dual enzymatic activity: peroxidase (POD)‐like and glutathione peroxidase (GPx)‐like activities for antimicrobial effects. Additionally, CuMPBA disrupts *Streptococcus pneumoniae (Sp)* metabolism by interfering with thiamine utilization, amino acid synthesis, DNA processes, and energy production. In burn‐ALI mice with secondary pneumonia, CuMPBA restored lung architecture, suppress TNF‐α/IL‐1β/IL‐6 levels, and modulated inflammatory pathways by activating Nrf2 while inhibiting NF‐κB. These synergistic mechanisms (precise bactericidal action combined with toxin neutralization) establish CuMPBA as a promising dual‐target therapeutic strategy for complex burn‐associated ALI. The multifunctionality of nanozymes addresses both infection control and inflammation resolution, offering new potential for managing this severe pathological condition.

## Graphical abstract

A novel copper nanocluster coated with 4‐mercaptobenzeneboronic acid (CuMPBA) is developed to target bacterial polysaccharides for addressing complicated acute lung injury (ALI) caused by severe burns and early pulmonary infections. CuMPBA exhibits dual anti‐inflammatory and antibacterial mechanisms by: (a) binding and degrading toxic bacterial components (LPS/LTA) through borate ester bonds, (b) killing bacteria via distrupting its thiamine metabolism, and (c) blocking the NF‐κB pathway, activating the Nrf2 pathway and scavenging excessive ROS to mitigate oxidative stress. This multifunctional nanomaterial demonstrates efficacy in preventing and treating ALI by scavenging bacterial polysaccharides, reducing inflammation, and secondary bacterial pneumonia.

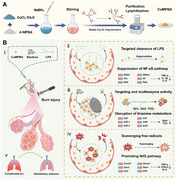

## 1 Introduction

Severe burns, as a critical traumatic condition, not only cause local skin and soft tissue damage but also trigger systemic immune responses and organ dysfunction.^[^ ^1^ ^]^ ALI and associated secondary pulmonary infections are the most common and life‐threatening complications following burns, significantly affecting patient prognosis.^[^ ^2^ ^]^ Clinical studies suggest that approximately 20%–56% of patients with severe burns may develop ALI reported in clinical studies, with a mortality rate significantly higher than that of burn patients without lung injury.^[^ ^3^ ^]^ As the burn area and depth increase, immune system dysregulation, pulmonary bacterial infections, and the release of bacterial toxins (such as LPS and LTA) exacerbate the inflammatory response, making ALI increasingly inevitable.^[^ ^4^ ^]^ Therefore, preventing and treating complex ALI induced by burns is crucial for improving the survival rate of burn patients. Currently, common clinical treatment methods, in addition to respiratory and fluid support therapy, include antibiotics targeting Gram‐positive bacteria (such as vancomycin) and anti‐inflammatory drugs (such as dexamethasone).^[^ ^5^ ^]^ However, this simultaneous antimicrobial and anti‐inflammatory treatment regimen often fails to achieve a balance, increasing the burden on various organ functions and further extending the effective dosage and treatment duration of antibiotics and steroids. As a result, finding a novel therapeutic approach that not only provides antibacterial activity but also neutralizes bacterial toxins to reduce the immune response is essential.

In recent years, metal nanomaterials have garnered widespread attention due to their diverse catalytic activities and high compatibility.^[^ ^6^ ^]^ Studies have shown that Fe‐curcumin nanoenzymes and Pd‐loaded covalent organic frameworks combined with near‐infrared light can eliminate intracellular reactive oxygen species (ROS) and inhibit inflammation in ALI.^[^ ^7^ ^]^ Gold nanoparticles (AuNPs) can selectively reduce the production of ROS and pro‐inflammatory cytokines in spleen cells induced by LPS.^[^ ^8^ ^]^ Copper‐based nanoenzymes exhibit multiple enzyme activities, such as POD, oxidase (OXD), catalase (CAT), superoxide dismutase (SOD) and GPx enzyme activities, playing a significant role in bacterial eradication and tissue inflammation regulation.^[^ ^6^, ^9^ ^]^ Furthermore, copper, as an essential element for the human body, plays a critical role in normal tissue cells and has relatively good biocompatibility under controlled conditions.^[^ ^10^ ^]^ Our team's recent research has discovered that CuCo_2_O_4_ nanoflowers and CuGA‐VAN not only possess enhanced multi‐enzyme activities but can also target proteins or amino groups on the bacterial cell wall, precisely inducing effects similar to copper‐mediated bacterial cell death.^[^ ^11^ ^]^ Building on previous research, the development of copper‐based nanomaterials with multiple enzyme activities to address bacterial infections, and regulate pulmonary tissue inflammation to treat ALI, is feasible.

Copper‐based nanomaterials offer numerous advantages, but their practical application is limited by several factors, such as the tendency of pure copper nanoparticles to aggregate, oxidize easily, and lack targeting capabilities. 4‐Mercaptobenzoic acid (4‐MPBA) is an organic boron compound that combines the functional groups of phenylboronic acid and a thiol (‐SH) group.^[^ ^12^ ^]^ The boronic acid group exhibits excellent biochemical activity, enabling it to target and bind to the cis‐diol groups at the 1,2‐ and 5,6‐positions of bacterial surface polysaccharides, forming a stable cyclic boronate ester structure.^[^ ^13^ ^]^ It can also interact with proteins and nucleic acids released from lysed bacteria, helping to target and neutralize bacterial toxins. The thiol group has strong reductive properties, forming covalent bonds with metal nanoparticles to stabilize them, and enhancing the nanoparticle's ability to scavenge free radicals under oxidative stress conditions.^[^ ^14^ ^]^ Research has shown that 4‐MPBA‐functionalized gold nanoparticles (4‐MPBA‐AuNPs) can bind to the teichoic acid and DNA of *vancomycin‐resistant enterococci (VRE)*.^[^ ^15^ ^]^

**Scheme 1:**
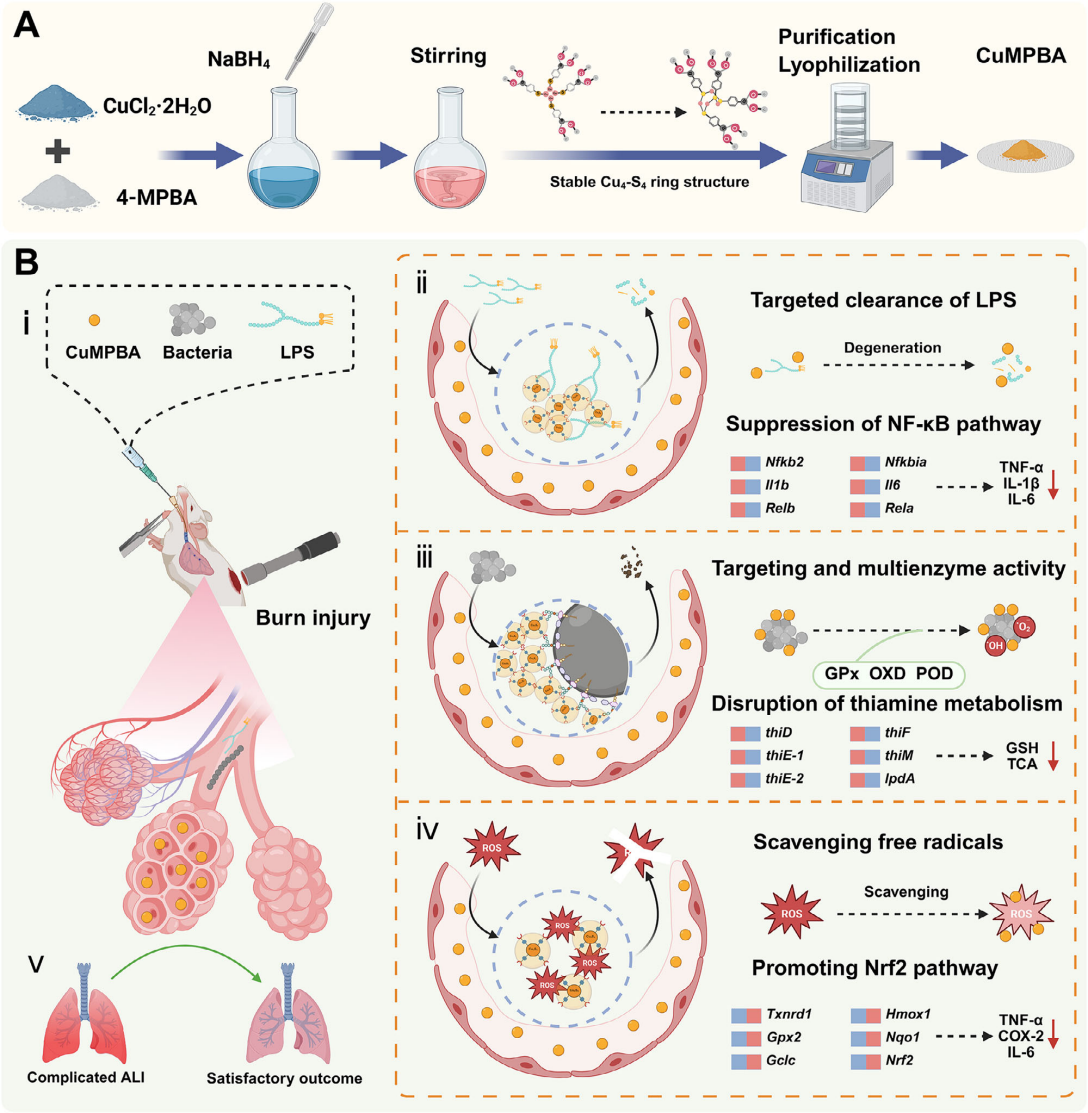
Illustration of CuMPBA and proposed mechanisms for complicated ALI. A) Synthesis process of CuMPBA. B) Multiple mechanisms of CuMPBA function: (i) the animal model of complicated ALI; (ii) targeting effects of LPS and suppression of inflammation; (iii) targeting and multienzyme activity against bacteria; (iv) scavenging free radicals and promoting of anti‐oxidative stress; (v) effectiveness from complicated ALI to satisfactory outcome.

In this study, a simple one‐pot method was used to synthesize copper nanozymes coated with 4‐MPBA. When CuMPBA targets bacterial surface polysaccharides, copper ions are rapidly released and accumulate around the bacteria. At low concentrations, this provides sufficient antibacterial activity and neutralizes bacterial toxins, while also scavenging free radicals produced by tissue oxidative stress. Further, in vivo experiments explored its mechanism for preventing complex ALI, and the findings suggest that it primarily functions through two main mechanisms: 1) CuMPBA neutralizes bacterial toxins by targeting and binding with LPS, interrupting the LPS‐activated NF‐κB pathway. Meanwhile, the locally released copper ions activate the Nrf2 pathway, enhancing the local lung tissue's ability to respond to ALI. 2) CuMPBA targets the LTA on the surface of *Sp*, disrupting the bacterial cell wall, which leads to the accumulation of copper ions around the bacteria. These copper ions catalyze the production of ROS, deplete the bacteria's glutathione (GSH), and interfere with thiamine metabolism, resulting in bacterial death. Overall, CuMPBA, as a novel nanomaterial with targeted antibacterial activity, bacterial toxin neutralization, and excellent biocompatibility, effectively alleviates bacterial infections, interrupts inflammation pathways, and prevents and treats complex ALI induced by burns. It represents a scientifically effective and promising strategy for the development of nanomedicines (**Scheme** **1**).

## 2 Results and Discussion

### 2.1 Synthesis and Characterization of CuMPBA

**Figure 1:**
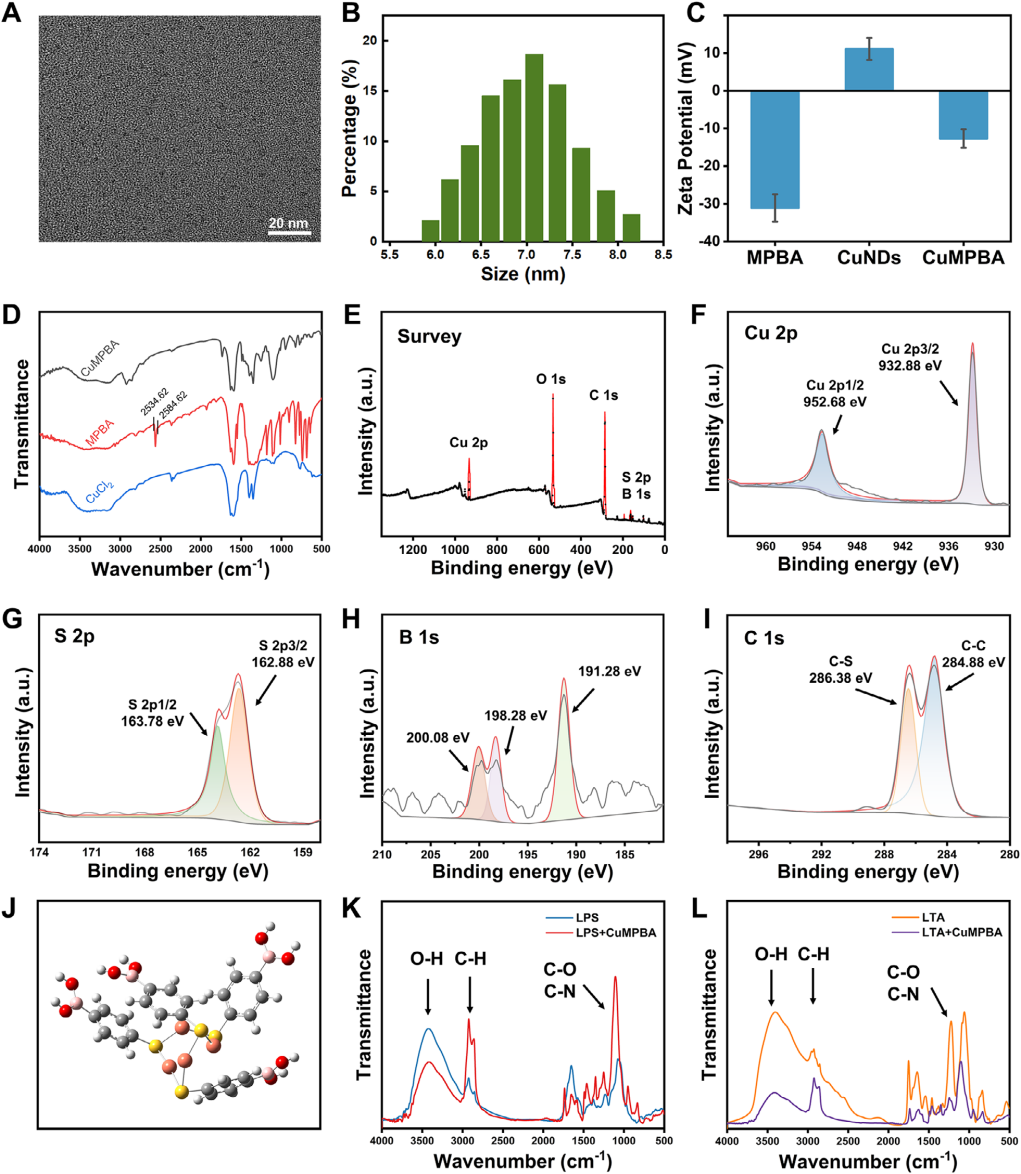
Characterization of CuMPBA. A) TEM image of CuMPBA; B) DLS image of CuMPBA; C) zeta potential test of CuMPBA; D) FTIR spectrum of CuMPBA; E–I) XPS spectrum of CuMPBA; J) calculated structure of CuMPBA; K, L) FTIR spectrum of CuMPBA+LPS/LTA.

CuMPBA was synthesized via the use of NaBH_4_ for in situ reduction modified from the one‐pot synthesis method of Au_44_(MBA)_18_.^[^ ^15^ ^]^ Transmission electron microscopy (TEM) images revealed that CuMPBA has a uniform spherical shape with good dispersibility and an average particle size of 3.07 ± 0.66 nm (**Figure** **1**A), whereas DLS measurements in aqueous solution revealed a size of 7.36 ± 0.58 nm (Figure 1B). Zeta potential measurements revealed values of −27.80 ± 3.81 mV for MPBA, 11.09 ± 2.93 mV for copper nanodots (CuNDs), and −12.66 ± 2.47 mV for CuMPBA (Figure 1C). The FTIR spectrum of MPBA revealed a thiol group (–SH) stretching between 2534.62 cm^−1^ and 2584.62 cm^−1^, with characteristic benzene ring vibrations between 1000–1600 cm^−1^ and boronic acid group peaks at 1200–1400 cm^−1^. In CuMPBA, the thiol group peak disappeared, whereas the benzene ring and boronic acid group peaks remained, suggesting thiol coordination with Cu (Figure 1D). XPS confirmed the presence of Cu, O, S, C, and B, with Cu 2p peaks at 932.88 eV and 952.68 eV, indicating the presence of Cu(I) and the absence of Cu(II). The S 2p and B 1s peaks suggested stable Cu‐S bonds and an unaltered boronic acid group environment (Figure 1E–I). The XRD results revealed an amorphous structure for CuMPBA (Figure S1A, Supporting Information), whereas HRMS revealed a peak cluster corresponding to tetranuclear copper, with a molecular weight of 822 Da (Figure S1B, Supporting Information). Density functional theory (DFT) calculations confirmed the most stable conformation of CuMPBA as an S‐Cu eight‐membered ring with the phenylboronic acid group extending outward (Figure 1J).^[^ ^16^ ^]^ FTIR analysis of LPS and LTA revealed characteristic polysaccharide vibrations, with both exhibiting O‐H, C‐H, and C = O/C‐N peaks, suggesting that CuMPBA forms hydrogen bonds or coordination bonds with the hydroxyl groups on LPS and LTA (Figure 1K,L). Together, these data confirmed that Cu was associated with 4‐MPBA via Cu‐S bonds.

### 2.2 Multienzyme Activity, Release of Copper Ions, and Target Performance of Bacterial Polysaccharides

**Figure 2:**
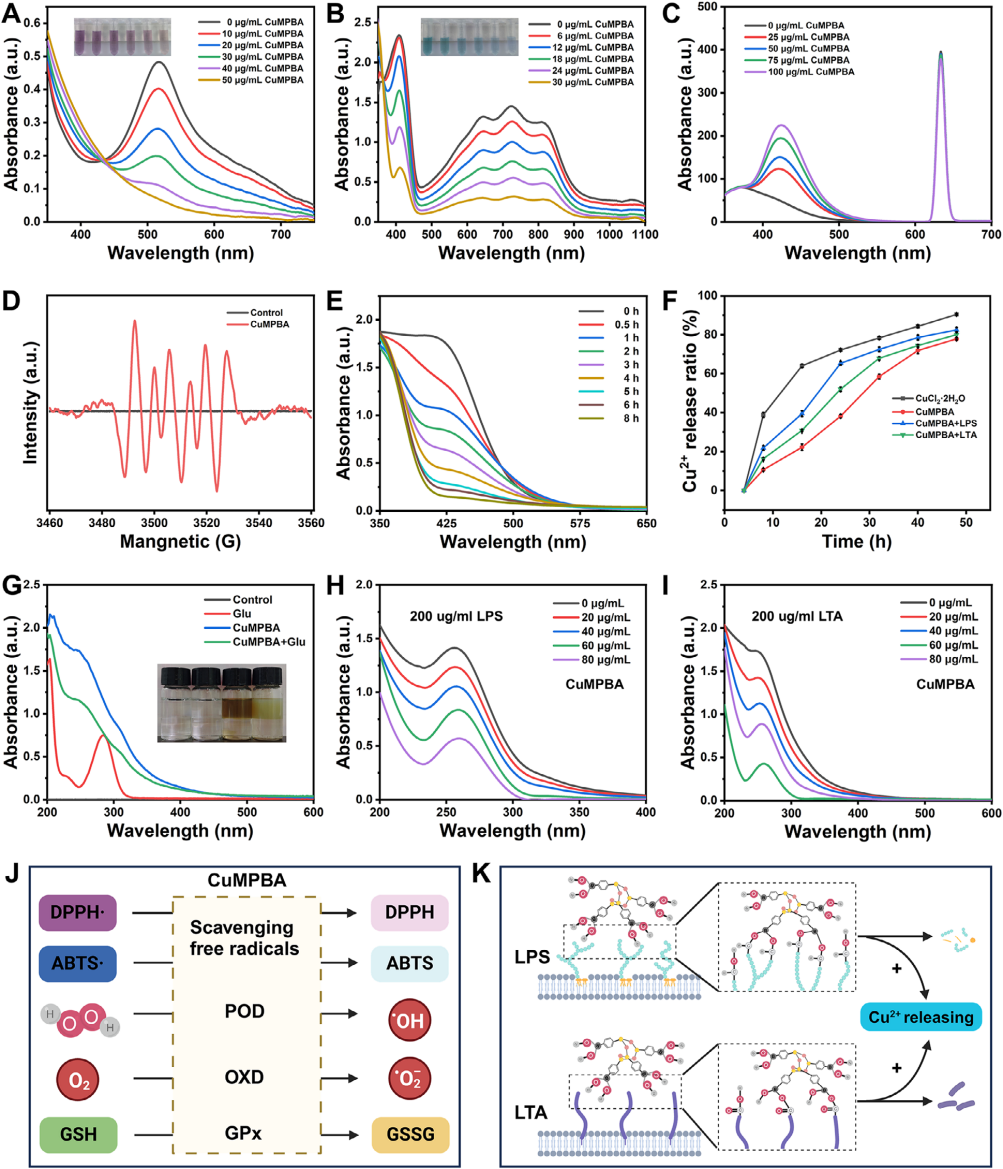
Multienzyme activity, release of copper ions and target performance of bacterial polysaccharides. A) DPPH; B) ABTS; C) PTA; D) ESR of CuMPBA; E) Consumption of GSH by CuMPBA; F) Cu^2+^ releasing from CuMPBA; G–I) Target performance of bacterial polysaccharides; J) Schematic diagram of CuMPBA in vitro enzymatic activity; K) Schematic diagram of CuMPBA binding and clearance with LPS and LTA.

Copper‐based nanomaterials, which use copper atoms as catalytic centers, exhibit various enzyme‐like activities. The in vitro enzyme‐like functions of CuMPBA were examined to evaluate its biomedical potential. 2,2‐Diphenyl‐1‐picrylhydrazyl (DPPH) assays revealed that as the CuMPBA concentration increased, the absorbance at 517 nm gradually decreased, indicating a concentration‐dependent free radical scavenging ability (**Figure** **2**A). Similarly, 2,2′‐Azinobis (3‐ethylbenzothiazoline‐6‐sulfonic acid) (ABTS) assays revealed reduced absorbance at 734 nm with higher CuMPBA concentrations, confirming its capacity to eliminate ABTS•+ and validating its antioxidant activity (Figure 2B). When Phthalic acid (PTA) was used to assess POD‐like activity, CuMPBA treatment led to a significant increase in absorbance at 424 nm, demonstrating its ability to decompose H_2_O_2_ into hydroxyl radicals (Figure 2C). Electron spin resonance (ESR) spectroscopy further confirmed this ROS‐generating OXD‐like enzyme activity (Figure 2D). In o‐phenylenediamine (OPD) assays, CuMPBA combined with H_2_O_2_ resulted in greater absorbance than the controls did, with the intensity increasing with increasing CuMPBA and H_2_O_2_ concentrations, demonstrating concentration‐dependent peroxidase‐like activity (Figure S2A–C, Supporting Information). Similarly, in the 3,3′,5,5′‐tetramethylbenzidine (TMB)‐based colorimetric tests, CuMPBA increased peroxidase‐like activity in a concentration‐dependent manner (Figure S2D–F, Supporting Information). In the GSH consumption assay using 5thio‐2‐nitrobenzoic acid (DTNB), the absorbance at 425 nm progressively decreased, dropping to half after 2 h, indicating strong GSH‐Px activity (Figure 2E).

To examine copper ion release, ICP analysis revealed that while CuCl_2_·2H_2_O released all the copper within 18 hours, only 60% of the CuMPBA was released within 48 hours. However, when LPS or LTA was present, the release reached ≈80%, indicating accelerated copper ion release when CuMPBA interacted with bacterial polysaccharides (Figure 2F). Binding ability was further confirmed using glucose as a model polysaccharide: after 20 minutes of reaction with CuMPBA, visible flocculation was observed, unlike in the CuMPBA‐only group, suggesting the role of MPBA in binding (Figure 2G,I). Finally, the ability of CuMPBA to clear LPS and LTA was concentration‐dependent, as shown by the decreased absorbance in the supernatant. Overall, CuMPBA has multiple enzyme‐like activities, including POD‐like and GSH‐Px activities, which together increase ROS‐based bacterial killing (Figure 2J). The MPBA coating enables controlled copper ion release, which accelerates upon interaction with LPS/LTA (Figure 2K). Moreover, CuMPBA specifically targets bacterial polysaccharides and effectively scavenges free radicals, making it a promising agent for antibacterial and anti‐inflammatory therapies.

### 2.3 In Vitro Antibacterial Efficiency Assessment

**Figure 3:**
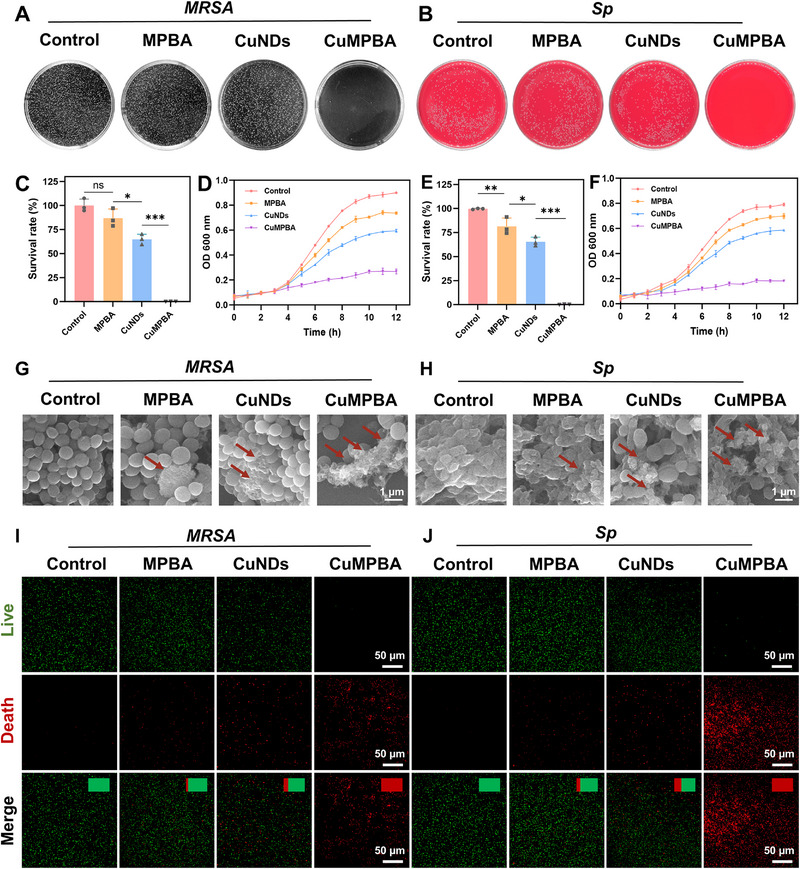
In vitro antibacterial efficiency assessment. A, B) Photographs of bacterial colonies to verify the antibacterial performance of CuMPBA against *MRSA* and *Sp*. C, E) Statistical analysis of the anti‐bacteria effects from (A, B). D, F) The antibacterial performance of CuMPBA was tested via the bacterial growth curve method against *MRSA* and *Sp*. G, H) SEM was used to verify the antibacterial performance of CuMPBA against *MRSA* and *Sp* (arrows show the destruction of bacterial structure). I, J) Bacterial viability staining was used to verify the antibacterial performance of different components of CuMPBA against *MRSA* and *Sp*. Data are presented as mean ± SD (n = 3). *P < 0.05, **P < 0.01, ***P < 0.001; ns, not significant (P > 0.05).

Building on previous findings regarding the enzyme‐like activity and targeting properties of CuMPBA, its in vitro antibacterial effects were further tested. Bacterial viability assay using clinical bacteria, including *methicillin‐resistant Staphylococcus aureus* (*MRSA*), *Sp* (gram‐positive), and *Escherichia coli* (*E. coli*) (gram‐negative), was performed (Figure S3A–F, Supporting Information). The results showed that CuMPBA exhibited concentration‐dependent antibacterial effects, with better efficacy against gram‐positive bacteria. Even at 200 µg mL^−1^, CuMPBA did not completely eradicate *E. coli*, likely due to the thicker cell wall of gram‐negative bacteria, which hinders the penetration of copper ions and ROS. *MRSA* showed lower sensitivity, whereas *Sp* exhibited higher susceptibility to CuMPBA. Therefore, *MRSA* and *Sp* were selected for further studies on the antibacterial mechanisms of CuMPBA. The antibacterial activities of the individual components were also compared, and it was found that MPBA alone had weak antibacterial effects, likely due to its ability to bind to bacterial surfaces. The CuNDs also exhibited relatively weak antibacterial effects (**Figure** **3**A–F). However, CuMPBA effectively killed both *MRSA* and *Sp*. SEM analysis confirmed that MPBA bound to the bacterial surface without significant morphological changes, whereas CuMPBA caused bacterial shrinkage and rupture (Figure 3G,H). Further verification was performed via a bacterial viability assay. After the bacteria were coincubated with the components, live/dead staining (N01/PI) was used, with live bacteria emitting green fluorescence and dead bacteria emitting red fluorescence. At 120 µg mL^−1^, CuMPBA killed almost all the *MRSA*, while the other components were ineffective. At 20 µg mL^−1^, CuMPBA nearly eradicated *Sp*, whereas the other components failed to kill Sp effectively (Figure 3I,J). These results align with previous findings. Thus, this study confirms the in vitro antibacterial effects of CuMPBA, leveraging its ability to target bacterial polysaccharides and release copper ions. It is speculated that the MPBA coating may enhance the solubility and binding efficiency of MPBA to bacteria in aqueous environments. Through this targeted action, CuMPBA efficiently releases copper ions and catalyzes hydroxyl radical production around bacteria, leading to effective antibacterial activity.

### 2.4 In Vitro Evaluation of Antibiofilm Efficiency

**Figure 4:**
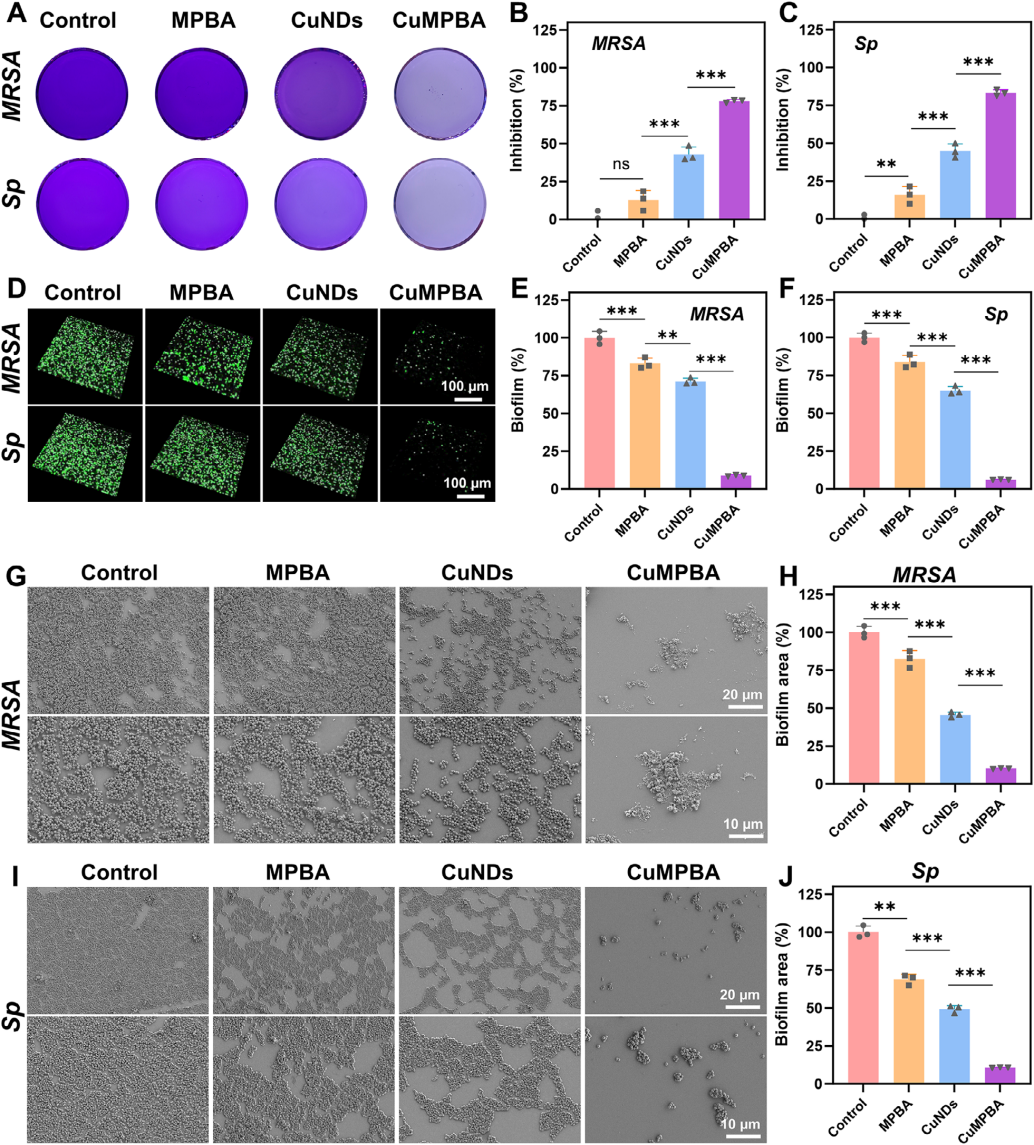
In vitro inhibition evaluation of antibiofilm efficiency. A) Verification of the biofilm inhibition effects of different components of CuMPBA on *MRSA* and *Sp* via crystal violet staining. B, C) Statistical analysis of the anti‐biofilm effects from (A). D) Fluorescence staining images and 3D reconstruction of the effects of biofilm inhibition on *MRSA* and *Sp*. E, F) Statistical analysis of the results from (D). G, I) SEM analysis of the biofilm inhibition effects of different components of CuMPBA on *MRSA* and *Sp*. H, J) Statistical analysis of the results from (G, I). Data are presented as mean ± SD (n = 3). *P < 0.05, **P < 0.01, ***P < 0.001; ns, not significant (P > 0.05).

In ALI caused by burns with concurrent pulmonary infection, bacterial infection often arises as a secondary complication due to systemic stress and lung inflammation. This type of infection resembles community‐acquired pneumonia, particularly lobar pneumonia, where the infecting bacteria are typically opportunistic lung colonizers, mainly gram‐positive bacteria such as *Staphylococcus pneumoniae* (*Sp*). These infections progress over time, as bacterial infection and proliferation require a period for biofilm formation, which helps bacteria resist immune system attacks and provides some antibiotic resistance. Therefore, preventing and disrupting biofilm formation is crucial. In biofilm inhibition experiments, crystal violet staining revealed that CuMPBA effectively inhibited biofilm formation, with approximately 75% and 80% inhibition of *MRSA* and *Sp*, respectively (**Figure** **4**A–C). 3D biofilm fluorescence staining (Figure 4D–F) and SEM imaging (Figure 4G–J) further confirmed that, compared with the compact biofilms in the control group, the CuMPBA‐treated biofilms had significantly smaller areas and less dense structures. Similar results were observed in biofilm disruption experiments, where CuMPBA achieved approximately 76% and 79% disruption rates for *MRSA* and *Sp*, respectively (Figures S4–S6, Supporting Information). In contrast, compared with CuMPBA, CuNDs, which lack targeting properties, inhibited and disrupted 49% and 39% of *Sp* biofilms, respectively, indicating weaker penetration and reduced efficacy.

These findings suggest that CuMPBA targets and disrupts biofilms through several mechanisms. First, the bacterial targeting ability of CuMPBA allows it to bind tightly to the bacterial cell wall on biofilm surfaces, catalyze ROS production, and release ions in the biofilm environment. Second, the POD‐like catalytic activity of CuMPBA converts hydrogen peroxide (H_2_O_2_) into hydroxyl radicals (·OH), which accumulate on the biofilm surface and disrupt the biofilm structure by damaging bacterial membranes and walls. Third, the GPx of CuMPBA consumes GSH in the biofilm microenvironment, enhancing its ability to clear biofilms. These findings highlight the potential of CuMPBA in preventing or treating biofilm‐associated infections caused by lung colonizers in vivo.

### 2.5 Mechanisms by Which CuMPBA Targets Bacterial Polysaccharides

The most stable structural conformation of CuMPBA, featuring a Cu‒S eight‐membered ring at its core, was previously identified. The O‐antigen polysaccharide structure was subsequently constructed on the outer side of the LPS, along with the N‐acetyl‐D‐glucosamine structure on the outermost side of the LTA. A reaction simulation revealed that the B(OH)_2_ group of CuMPBA undergoes condensation with the vicinal diol group of the polysaccharide, forming a stable boronate ester structure (five‐ or six‐membered) and releasing water. The reaction equation is as follows:

(1) $$\begin{array}{cc} & \mathrm{CuMPBA}-B{\left(\mathrm{OH}\right)}_{2}+R-\mathrm{CHOH}-\mathrm{CHOH}-R^{\prime }\\ & \;\to \mathrm{CuMPBA}-B\left(\mathrm{OR}-\mathrm{CHOH}-{\mathrm{OR}}^{\prime }\right)\;+\;H_{2}O \end{array}$$

**Figure 5:**
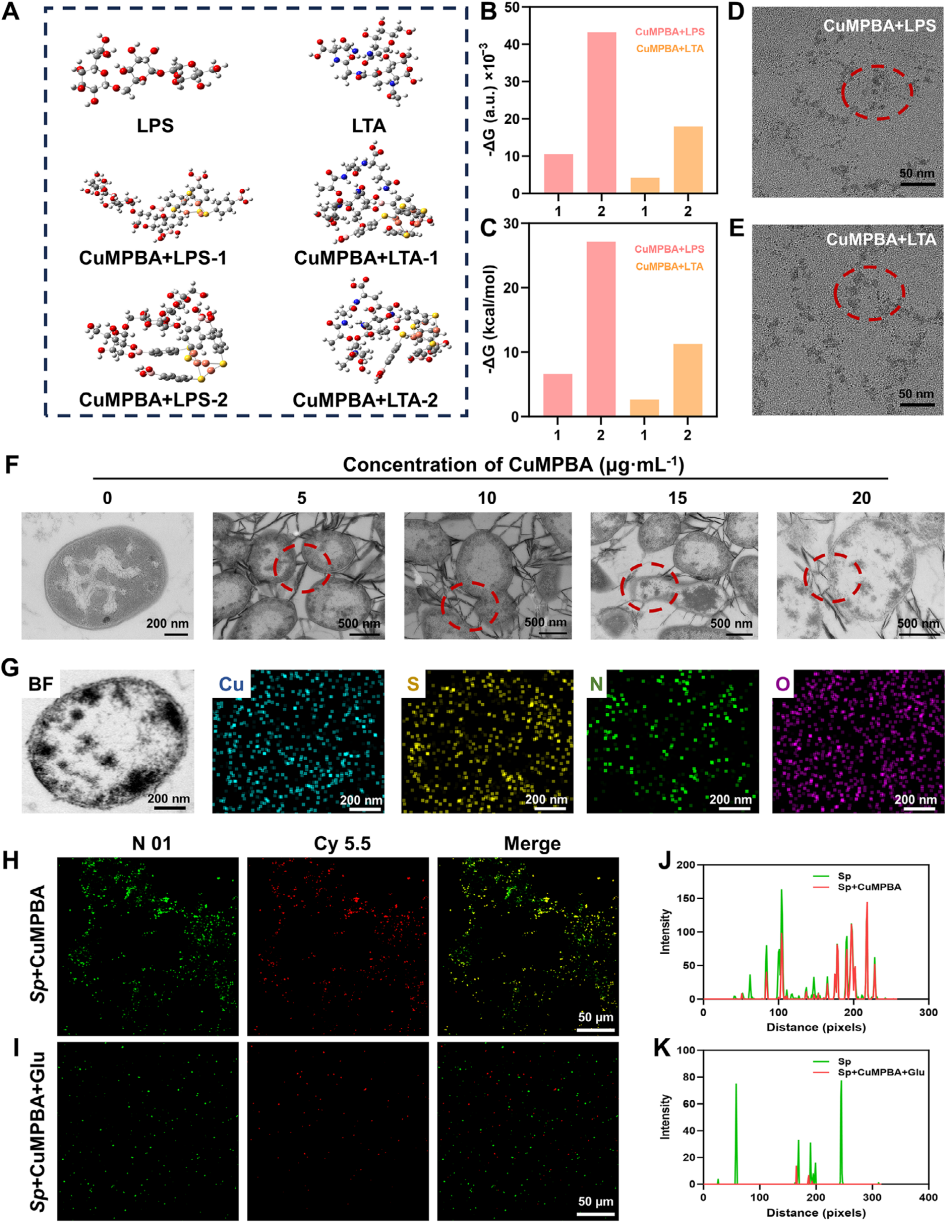
In‐depth study of the mechanism by which CuMPBA affects bacterial polysaccharides. A) Stable conformations of CuMPBA and the two most stable conformations when it is bound with LTA and LPS. B, C) Thermodynamic simulation calculations of the four conformations in (A). D, E) Morphology observed via TEM after CuMPBA bound to LPS and LTA (red circles show the aggregated structures of CuMPBA). F) TEM observation of the disruption of *Sp* by CuMPBA. G) TEM‐EDS mapping was used to observe the elemental distribution around dead bacteria. H, I) Colocalization experiments of CuMPBA and *Sp*, where (H) represents untreated CuMPBA+*Sp* and (I) represents CuMPBA+*Sp* treated with glucose. J, K) Statistical analysis of the colocalization fluorescence results for (H, I).

The two most stable reaction conformations were analyzed, and their ΔG (a.u.) and ΔG (kcal mol^−1^) values were compared. The negative ΔG values for the CuMPBA‐LPS and CuMPBA‐LTA reactions suggest that both reactions are spontaneous and thermodynamically favorable. Among the two conformations for CuMPBA binding with LPS or LTA, the second conformation had a more negative ΔG, indicating greater stability. Notably, the ΔG for CuMPBA binding with LPS was more negative, especially for the second pathway (ΔG = −27.13 kcal mol^−1^), suggesting that CuMPBA prefers to react with LPS over LTA (**Figure** **5**A–C). Next, the morphology of CuMPBA in the presence of LPS or LTA was observed via TEM. The originally uniform spherical shape of CuMPBA changed to an aggregated, dendritic form, indicating that a reaction with LPS or LTA altered its structure and further TEM‐EDS mapping confirmed the Cu and S enrichment derived from CuMPBA (Figure 5D,E; Figure S7, Supporting Information). The products cocultured with CuMPBA and *Sp* were also examined via TEM. In the control group, the *Sp* morphology was intact, with clear cell walls and membranes. However, in the CuMPBA‐treated *Sp* group, CuMPBA aggregated around the bacteria into needle‐like structures. As the CuMPBA concentration increased from 0–20 µg mL^−1^, TEM images revealed that CuMPBA interacted with the bacterial cell wall, causing bacterial rupture, leakage of the cellular contents, and complete breakdown of the cell wall and membrane, leading to bacterial death (Figure 5F). TEM‐EDS mapping further confirmed copper (Cu) and sulfur (S) accumulation around the dead bacteria, with CuMPBA invading the bacteria and binding to the cellular contents (Figure 5G). Fluorescence colocalization techniques were used to confirm the targeting ability of CuMPBA. CuMPBA was labeled with Cy5.5 (red), and *Sp* was stained with N01 (green). In the CuMPBA+*Sp* group, red and green fluorescence significantly overlapped, indicating that CuMPBA targeted the bacterial surface (Figure 5H,J). However, when glucose was added, the fluorescence density and overlap decreased, suggesting that CuMPBA targets bacterial surface polysaccharides (Figure 5I,K). These results collectively demonstrate that CuMPBA can effectively target bacterial surfaces through polysaccharide interactions, contributing to its antibacterial activity and bacterial destruction mechanisms.

### 2.6 Transcriptomic Analysis of CuMPBA Killing of *Sp*

**Figure 6:**
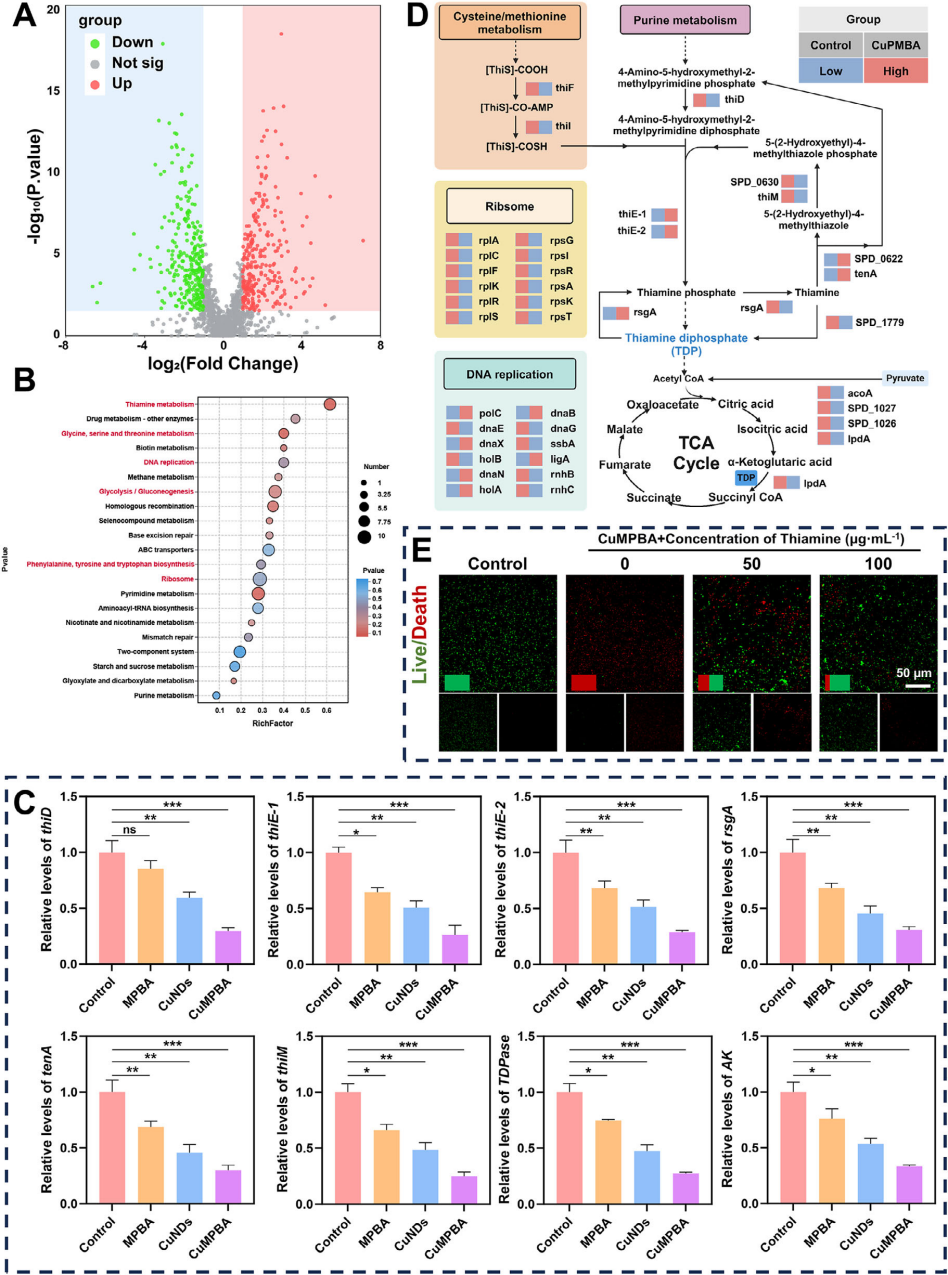
Transcriptomic analysis of CuMPBA killing of *Sp*. A) Volcano plot of the differentially expressed genes. B) GO enrichment of differential gene functions. C) qPCR results of typical genes involved in thiamine metabolism in bacteria (n = 3). D) Schematic diagram of the main antibacterial mechanism of CuMPBA against *Sp*. E) Live/dead fluorescence staining to evaluate the rescue effect of exogenous thiamine supplementation on bacteria (n = 3). P values were calculated for comparisons with the control group. Data are presented as mean ± SD. *P < 0.05, **P < 0.01, ***P < 0.001; ns, not significant (P > 0.05).

To further investigate the antibacterial mechanism of CuMPBA, transcriptional analysis was performed on *Sp* from both the control and the CuMPBA‐treated groups. The clustering heatmap revealed significant differences in gene expression, with 288 differentially expressed genes (DEGs) identified. A volcano plot revealed 157 upregulated and 131 downregulated DEGs, highlighting key genes involved (**Figure** **6**A). KEGG pathway analysis and heatmap analysis revealed that the DEGs were enriched predominantly in pathways related to thiamine metabolism, amino acid metabolism, glycolysis, and DNA replication (Figure 6B). Several genes involved in thiamine metabolism, including *thiD, thiE‐1, thiE‐2, rsgA, tenA, thiM, thiamine diphosphokinase (TDP), and adenylate kinase (AK)*, were significantly downregulated after CuMPBA treatment. Thiamine biosynthesis begins with 1‐(5′‐phospho‐ribosyl)‐5‐aminoimidazole and involves several enzymatic reactions catalyzed by these genes, ultimately converting this compound into thiamine diphosphate, which was crucial for glycolysis, the TCA cycle, and other energy metabolism processes (Figure 6D). Real‐time quantitative PCR (qRT‐PCR) was used to validate the expression of key thiamine biosynthesis genes. The results confirmed that the mRNA levels of *thiD, thiE‐1, thiE‐2, rsgA, tenA, thiM, TDP, and AK* were significantly reduced, which was consistent with the transcriptomic findings (Figure 6C). To further confirm that thiamine can reverse the antibacterial effects of CuMPBA, thiamine was added to the coculture medium supplemented with *Sp* and 50 µg mL^−1^ CuMPBA. As the thiamine concentration increased, the antibacterial effect of CuMPBA gradually diminished. At 100 µg mL^−1^ thiamine, the antibacterial effect was significantly reversed. Thiamine metabolism is closely linked to bacterial oxidative stress and energy metabolism (Figure 6E). These results suggest that CuMPBA inhibits thiamine biosynthesis, disrupting oxidative stress responses, energy metabolism, and protein synthesis and leading to bacterial death. CuMPBA also affects thiamine diphosphate synthesis by blocking thiamine metabolism, which disrupts pyruvate metabolism, glycolysis/gluconeogenesis, and the TCA cycle. This interference with bacterial energy metabolism and cell proliferation impairs the ability of bacteria to maintain energy and reproduce. The regulation of energy metabolism genes is key to the antibacterial and antibiofilm effects of CuMPBA. Furthermore, CuMPBA significantly impacts the bacterial nucleotide replication pathway, which is crucial for the response to oxidative stress. When external ROS damage DNA, bacteria can repair the damage and continue DNA replication. CuMPBA interferes with the genes responsible for DNA repair and replication, reducing the ability of bacteria to repair ROS‐induced DNA damage and thus decreasing bacterial survival.

Therefore, CuMPBA exerts its antibacterial effects on *Sp* by disrupting key metabolic pathways, such as thiamine biosynthesis, glycolysis, the TCA cycle, and nucleotide replication. This multifaceted inhibition weakens bacterial oxidative stress responses, energy metabolism, and DNA repair mechanisms, leading to bacterial death.

### 2.7 Evaluation of Cytotoxicity and Anti‐Inflammatory Activity in Vitro

The biocompatibility of CuMPBA with the lung epithelial cell line A549 was first evaluated via a CCK‐8 assay (Figure S8, Supporting Information). The results showed that CuMPBA was nontoxic at a concentration of 200 µg mL^−1^, which was confirmed by hemolysis tests (Figure S9, Supporting Information). In a scratch migration assay with HUVECs, 100 µg mL^−1^ CuMPBA effectively promoted cell migration (Figure S10, Supporting Information). Next, the protective effects of CuMPBA on cells were assessed in vitro. The ROS levels were analyzed via the use of 2′,7′‐dichlorofluorescin diacetate (DCFH‐DA), a probe that detects ROS by emitting green fluorescence upon oxidation. A549 cells were pretreated with various materials for 2 hours, exposed to 100 µg mL^−1^ LPS for 12 hours, and incubated with DCFH‐DA for flow cytometry analysis. The LPS‐treated group presented a shift in fluorescence distribution, indicating the accumulation of ROS (Figure S11A, Supporting Information). However, cells pretreated with CuMPBA only slightly shifted, suggesting that CuMPBA protected against LPS‐induced ROS overload. Confocal microscopy with DCFH‐DA and DAPI probes confirmed these findings. A549 cells without LPS treatment presented no significant green fluorescence, whereas those in the LPS group presented strong fluorescence. In the 50 µg mL^−1^ CuMPBA‐treated group, the green fluorescence intensity was reduced, and in the 100 µg mL^−1^ CuMPBA‐treated group, it was significantly decreased (Figure S11B, Supporting Information). These results indicate that CuMPBA inhibits the accumulation of ROS induced by LPS, with a more pronounced protective effect at higher concentrations. In brief, CuMPBA protected A549 cells from LPS‐induced oxidative stress in a dose‐dependent manner.

### 2.8 In Vivo Treatment with CuMPBA Eliminates Burn‐Induced ALI Caused by Pneumonia

**Figure 7:**
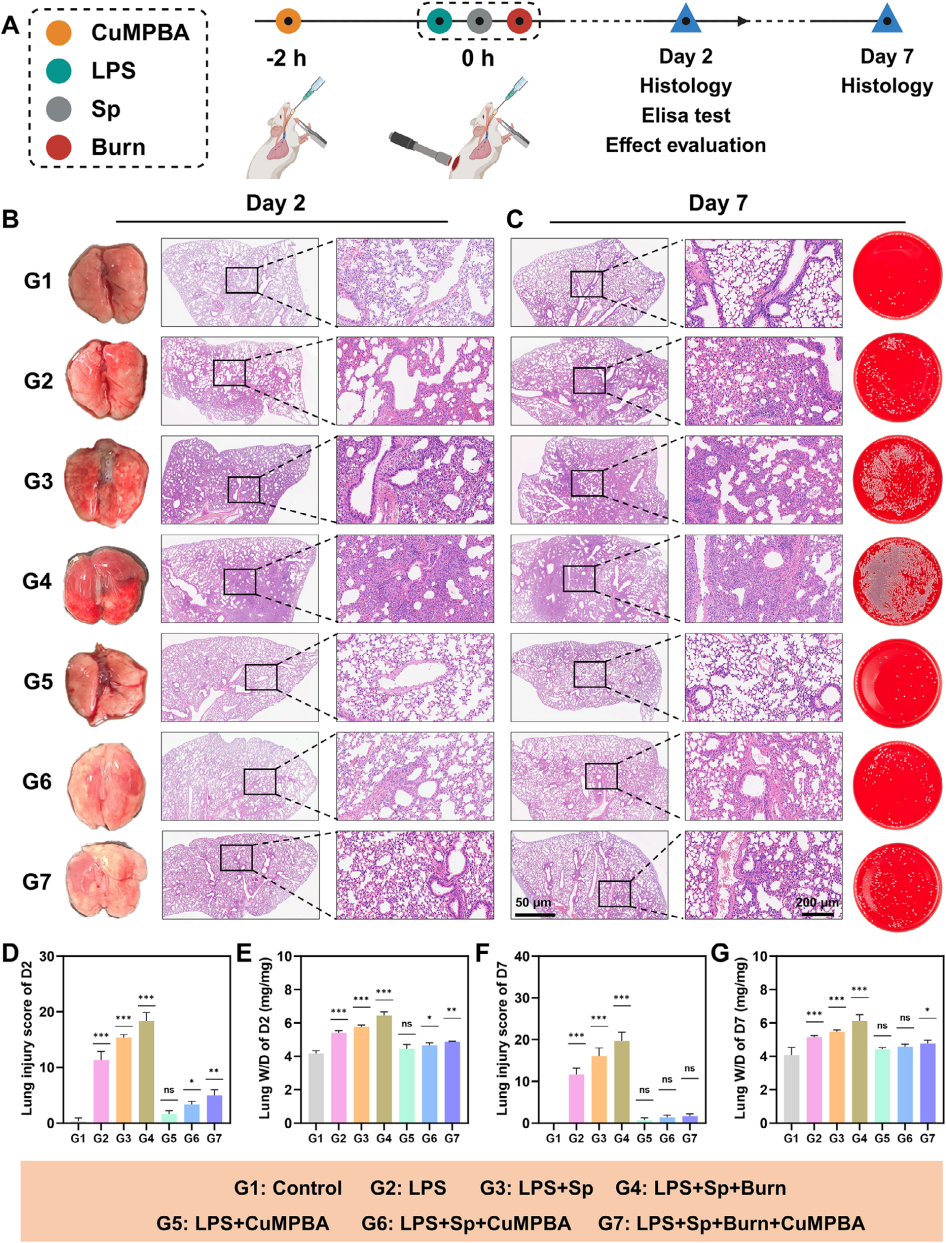
In vivo treatment with CuMPBA eliminates burn‐induced ALI caused by pneumonia. A) Flowchart of the animal experiment. B, C) Gross images of mouse lungs and HE‐stained sections on the second day for different groups, as well as HE‐stained sections and bacterial plating of bronchoalveolar lavage fluid on the seventh day (n = 5). D, E) Lung injury tissue scoring and the lung wet‒dry weight ratio on the second day (n = 5). F, G) Lung injury tissue scoring and the lung wet‒dry weight ratio on the seventh day (n = 5). P values were calculated for comparisons with the control group. Data are presented as mean ± SD. *P < 0.05, **P < 0.01, ***P < 0.001; ns, not significant (P > 0.05).

**Figure 8:**
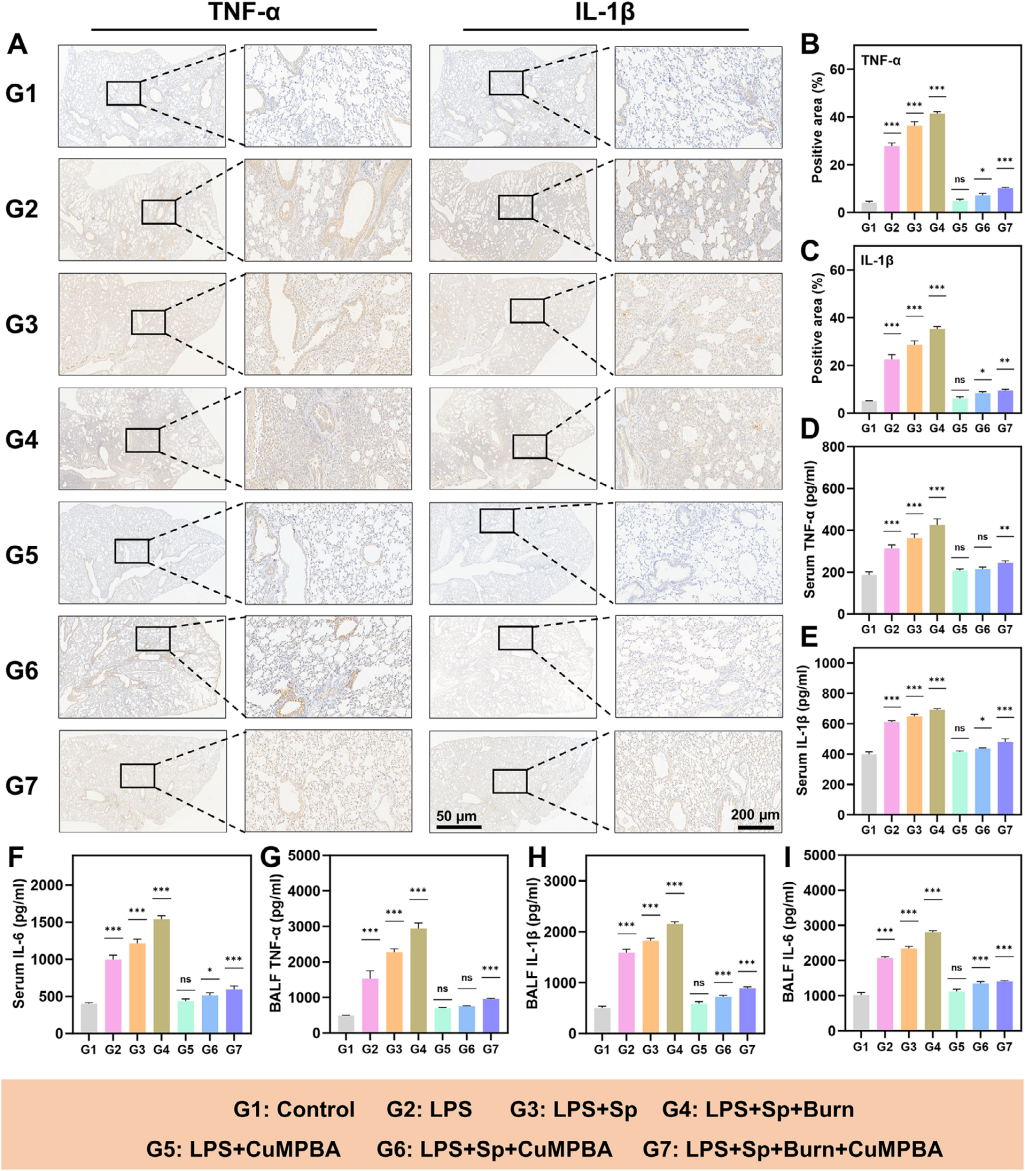
Transcriptomic analysis of the effects of CuMPBA treatment on ALI. A) Immunohistochemical sections of lung tissue on the second day. B, C) Statistical analysis of the positive area for the two indicators in (A). D–F) ELISA detection of TNF‐α, IL‐1β, and IL‐6 in the serum of the mice on the second day (n = 3). G–I) ELISA detection of TNF‐α, IL‐1β, and IL‐6 in bronchoalveolar lavage fluid from mice on the second day (n = 3). P values were calculated for comparisons with the control group. Data are presented as mean ± SD. *P < 0.05, **P < 0.01, ***P < 0.001; ns, not significant (P > 0.05).

As previously noted, in vitro studies confirmed that CuMPBA effectively targets LPS, exhibits antimicrobial activity, and protects against LPS‐induced oxidative stress. To further evaluate its therapeutic potential, complicated ALI mouse models were established with a schematic outlining the modeling and CuMPBA administration schedule (**Figure** **7**A). The mice were randomly assigned to seven groups: Control, LPS, LPS+*Sp*, LPS+*Sp*+Burn, LPS+CuMPBA, LPS+*Sp*+CuMPBA, and LPS+*Sp*+Burn+CuMPBA. All treatments, including LPS (5 mg kg^−1^) and *Sp* (8.5×10⁶ CFU), were administered via intratracheal injection (50 µL total). The Burn group received a third‐degree burn of skin on the back. CuMPBA (4 mg mL^−1^, 50 µL) was administered 2 hours before injury. Lung tissues were collected on days 2 and 7 for histological evaluation, and the levels of inflammatory cytokines (TNF‐α, IL‐1β, and IL‐6) in the serum and BALF were measured on day 7. By day 2, the mice in the LPS, LPS+*Sp*, and LPS+*Sp*+Burn groups presented symptoms, including lethargy, rough fur, reduced intake, and nasal/oral discharge. In contrast, CuMPBA‐treated mice maintained normal behavior and appearance. Lung histology revealed normal architecture in the control group, whereas the injury groups presented extensive inflammation, neutrophil infiltration, and alveolar damage (Figure 7B). CuMPBA treatment markedly improved lung structure and reduced inflammation, with significantly lower injury scores and lung wet/dry ratios (Figure 7D,E). By day 7, while inflammation slightly decreased in the untreated groups, prominent immune cell infiltration still occurred. CuMPBA‐treated mice showed further improvements, with reduced inflammation and lower bacterial counts in the BALF (Figure 7C,F,G). Immunohistochemistry revealed decreased expression of TNF‐α and IL‐1β in the lung tissues of the CuMPBA‐treated groups (**Figure** **8**A), and inflammatory marker levels (TNF‐α, IL‐1β and IL‐6) in the serum (Figure 8D–F) and BALF (Figure 8G–I) were consistently associated with reduced lung damage. Histological assessment of other organs and laboratory tests supported these findings (Figures S12–S14, Supporting Information). Hence, CuMPBA provides robust prevention and treatment across different ALI models, effectively mitigating inflammation and tissue damage, even under additional burn‐induced systemic stress, highlighting its potential in treating complex ALI scenarios.

### 2.9 CuMPBA Protects Lungs from ALI by Promoting the Nrf2‐Nqo1/Hmox1 Pathway and Interrupting the NF‐κB Pathway

**Figure 9:**
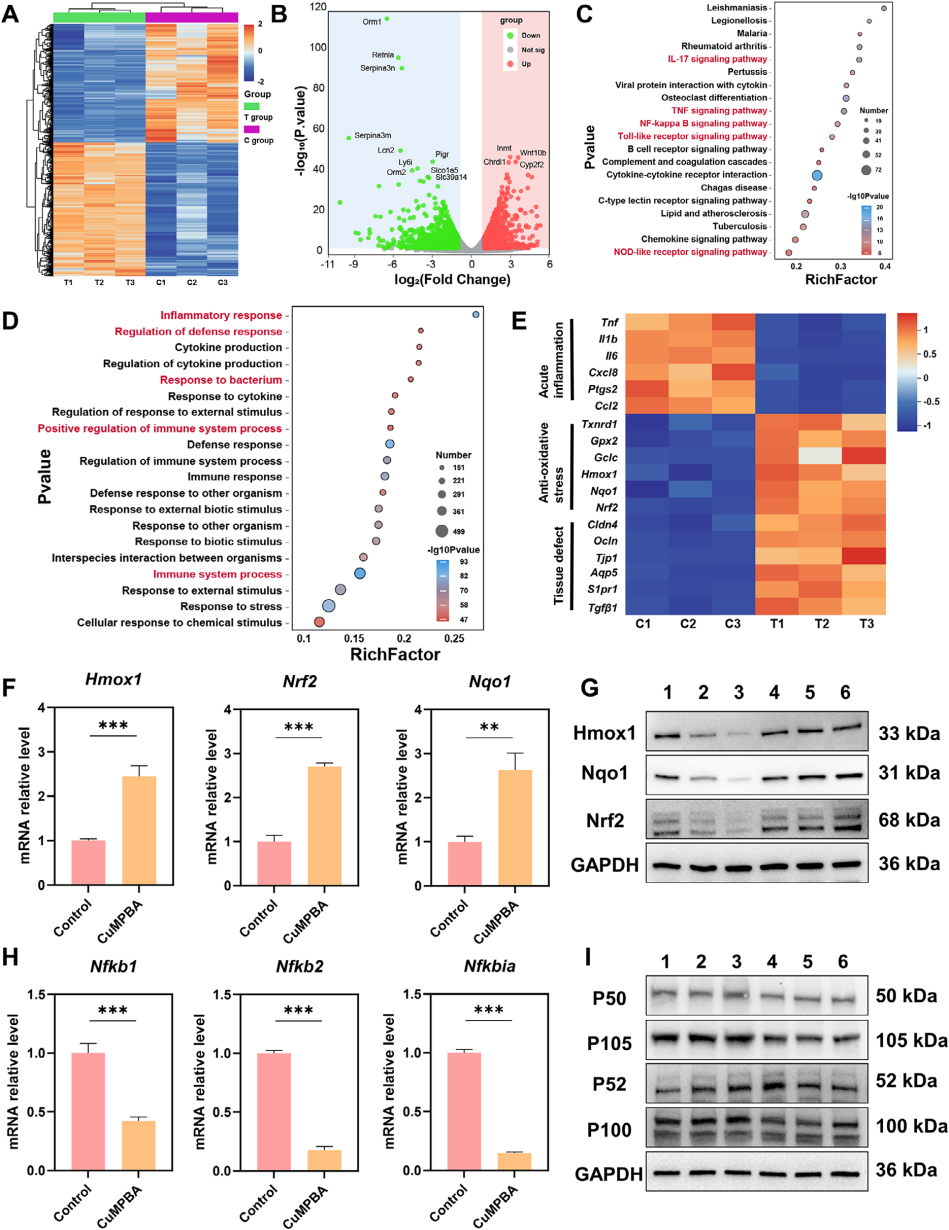
Transcriptomic analysis of the preventive and protective mechanisms of CuMPBA against ALI. A) Clustering heatmap of the sequenced genes. B) Volcano plot of the differentially expressed genes. C) KEGG enrichment of differential pathways. D) GO enrichment of differential gene functions. E) Heatmap showing the mechanisms of action of CuMPBA. F, H) qPCR of the key genes in the Nrf2 and NF‐κB pathways (Control: LPS+*Sp*+Burn, CuMPBA: LPS+*Sp*+Burn+CuMPBA) (n = 3). G, I) Western blotting of key genes in the Nrf2 and NF‐κB pathways (1: LPS, 2: LPS+*Sp*, 3: LPS+*Sp*+Burn, 4: LPS+CuMPBA, 5: LPS+*Sp*+CuMPBA, 6: LPS+*Sp*+Burn+CuMPBA) (n = 3). Data are presented as mean ± SD. *P < 0.05, **P < 0.01, ***P < 0.001; ns, not significant (P > 0.05).

To investigate the protective mechanism of CuMPBA in ALI, high‐throughput RNA sequencing (RNA‐seq) was performed to analyze differential gene expression in lung tissues from mice treated with CuMPBA and control mice on day 2 (D2). A total of 2657 differentially expressed genes (DEGs) were identified (log2‐fold change > 1.00; p‐value < 0.05). The heatmap of the transcriptomic data highlighted clear differences in gene expression between the two groups (**Figure** **9**A). A volcano plot revealed that 1412 genes were upregulated and that 1245 genes were downregulated after CuMPBA treatment (Figure 9B). KEGG pathway analysis revealed that the DEGs were significantly enriched in pathways such as the IL‐17, NF‐κB, TNF, Toll‐like receptor, chemokine signaling, cytokine–cytokine receptor interaction, and NOD‐like receptor signaling pathways (Figure 9C; Figure S15, Supporting Information). Gene Ontology (GO) enrichment analysis indicated that the DEGs were involved primarily in the inflammatory response, immune response, and response to external or bacterial stimuli (Figure 9D). Focusing on key genes related to acute inflammation, antioxidant stress, and tissue repair, heatmap visualization revealed significant changes in gene expression in these categories following CuMPBA treatment, suggesting that CuMPBA prevents and treats ALI by enhancing tissue antioxidant capacity, alleviating inflammation, and improving tissue resistance (Figure 9E). Based on the KEGG enrichment results, two key pathways were selected for transcriptional and protein‐level validation. qRT‐PCR and Western blot analyses revealed significant increases in the protein levels of Nrf2, Nqo1, and Hmox1 in the lung tissues of the CuMPBA‐treated groups (Figure 9F,G), indicating activation of the Nrf2‐Nqo1/Hmox1 pathway. Conversely, the protein levels of p100/52 and p105/p50 in the NF‐κB pathway were significantly reduced (Figure 9H,I). These findings suggest that CuMPBA exerts its protective effects by activating the Nrf2‐Nqo1/Hmox1 pathway and interrupting the NF‐κB pathway. Thus, CuMPBA prevents and treats ALI primarily by increasing the antioxidant capacity of lung tissue, mitigating inflammation, and increasing resistance to external stimuli and immune regulation, leading to effective therapeutic outcomes.

## 3 Conclusion

In summary, CuMPBA was successfully synthesized via a simple method aimed at preventing and treating ALI accompanied by secondary pulmonary infection caused by burns. CuMPBA demonstrated excellent POD‐like enzyme activity and GSH‐Px‐like activity, and it precisely targeted bacterial surface polysaccharides (LPS and LTA). Antibacterial experiments indicated that CuMPBA had good in vitro antibacterial and biofilm‐eliminating properties, whereas in vitro anti‐inflammatory experiments revealed that CuMPBA provided excellent protection against LPS‐induced oxidative stress in cells. Compared to other copper‐based nanomaterials, the ability of CuMPBA to bind to bacterial surface polysaccharides is crucial to its antibacterial efficacy. This binding allows ROS and Cu^2+^ to be released more rapidly and effectively in the microenvironment around bacteria, achieving optimal bactericidal effects while minimizing damage to normal mammalian cells. Moreover, the high specificity of CuMPBA for bacterial surface polysaccharides can significantly reduce LPS levels in lung tissue, eliminating the LPS content in advance to reduce oxidative stress damage and immune responses. This preventative effect may play a more critical role than treatment after inflammation has already occurred. Furthermore, transcriptomic sequencing of tissues and bacteria revealed that CuMPBA has excellent anti‐inflammatory effects by upregulating Nrf2 and downregulating the NF‐κB pathway. It also interferes with thiamine metabolism, amino acid metabolism, DNA repair, and energy metabolism in *Sp* colonizing the lungs. Overall, CuMPBA, which targets bacterial surface polysaccharide components and possesses broad‐spectrum catalytic properties, represents a promising biomedical strategy for treating the complex clinical conditions of ALI with secondary pulmonary infection induced by burns. Further research is required to validate clinical translation.

## Conflict of Interest

The authors declare no conflict of interest.

## Author Contributions

H.G. and M.W. contributed equally to this paper. H.G. performed conceptualization, methodology, data curation; wrote original draft; M.W. wrote and original draft preparation. H.X. performed theoretical and computational chemistry analysis; X.‐L.C. performed funding acquisition, experimental guidance, supervision; X.W. performed funding acquisition, wrote review and edited the original draft, supervision. All the authors have read and agreed to the published version of the manuscript.

## Supporting information



Supporting Information

## Data Availability

The data that support the findings of this study are available in the supplementary material of this article.
