# Supplementary material for: Phenylboronic Acid‐Modified Copper Nanozymes as Nanoscavengers of Bacterial Polysaccharides Causing Acute Lung Injury
Source: Adv Sci (Weinh). 2025 Sep 16;12(45):e13536. doi: 10.1002/advs.202513536 (PMC12677591; doi:10.1002/advs.202513536)
Supplement: Supplementary file 1 — Supporting Information [file ADVS-12-e13536-s001.docx]

**Supporting Information for**

Phenylboronic Acid-Modified Copper Nanoclusters as Nanoscavengers of Bacterial Polysaccharides Causing Acute Lung Injury

*Haojie Ge^#^, Min Wang^#^, Haijiao Xie, Xu-Lin Chen*, Xianwen Wang**

Dr. H Ge, Prof. X Chen, Prof. X. Wang

Department of Burns,

The First Hospital Affiliated of Anhui Medical University,

Anhui Medical University,

Hefei, Anhui 230032, P. R. China.

**E-mail:** [xianwenwang@ahmu.edu.cn](mailto:xianwenwang@ahmu.edu.cn) (X. Wang); **E-mail:** [okcxl@126.com](mailto:okcxl@126.com)(X. Chen)

Dr. M Wang, Prof. X. Wang

School of Biomedical Engineering,

Anhui Medical University,

Hefei 230032, China.

Mr. H Xie

Hangzhou Yanqu Information Technology Co., Ltd,

Hangzhou 310003, China.

#These authors contributed equally to this paper.

***Corresponding Authors**

# Materials and methods

## Synthesis of CuMPBA

The synthesis of CuMPBA followed a one-pot method, with the total volume of the reaction system being 10 mL of aqueous solution. The concentrations of the components were as follows: 4-MPBA, 15.4 mg; CuCl₂, 16.9 mg; triethylamine, 50 μL; Tween 80, 30 mg; and NaBH₄ aqueous solution (3 mg/mL), 2 mL. The 4-MPBA powder was first dispersed in pure water and subjected to ultrasonic treatment to obtain a homogeneous 4-MPBA dispersion. This dispersion is then mixed with the CuCl₂ solution, followed by the addition of triethylamine and Tween 80. NaBH₄ aqueous solution was added dropwise while stirring. After 2 h of reaction, the sample mixture was collected in a dialysis bag and dialyzed. After dialysis, the solution was freeze-dried to obtain the final CuMPBA.

## Characterization

The samples were analyzed morphologically via transmission electron microscopy (USA-FEI Talos F200X G2). High-angle annular dark-field scanning transmission electron microscopy (HAADF-STEM). Crystal structure information was obtained via X-ray powder diffraction (China-DX-2700BH). Fourier transform infrared (FTIR) spectroscopy was performed on the powder material (China-FOLI20). XPS was calibrated using the C1s peak at 284.8 eV as reference using a scanning X-ray microprobe (USA - Thermo Fisher + ESCALAB Qxi). Dynamic light scattering (DLS) and zeta potential were measured using a Brookhaven 90 Plus PALS analyzer. Electron spin resonance (ESR) spectroscopy was performed on a water mixture (Germany-Bruker EMXplus-6/1). The copper ions were tested via an inductively coupled plasma atomic emission spectrometer (ICP‒AES, China-EXPEC 6500).

## Peroxidase-like catalytic activity

To measure the peroxidase-like activity of the materials, the materials and hydrogen peroxide were tested in a PBS solution under neutral conditions containing TMB/OPD. The UV‒Vis absorbance of the coloring reaction mixture (652 nm for OxTMB and 492 nm for OPD) was measured and recorded for a given reaction time to assay the peroxidase-like activity of the materials.

## GSH consumption

GSH oxidation was detected via the Ellman method, and all the experiments were conducted in a dark environment. The yellow product 2-nitro-5-thiobenzoic acid was synthesized via the reaction of Ellman’s reagent 5,5'-dithiobis-(2-nitrobenzoic acid) (DTNB) and disulfide bonds (-SS-) in GSH. To investigate the time dependence of GSH consumption, CuMPBA was cultivated with GSH in PBS for different durations. The solution was then mixed with DTNB. The mixture was centrifuged, and the remaining suspension was analyzed via UV‒Vis absorption spectroscopy to identify GSH depletion.

## *In vitro* antimicrobial assay

The *in vitro* antimicrobial properties of the CuMPBA nanoflowers were determined via colonies counting to determine the number of CFUs on the plates. The gram-negative bacterial model was *E. coli*, and the gram-positive bacterial models were *MRSA* and *Sp*. First, the cryopreserved strains were resuspended in glycerol and incubated in broth for 8 h. After incubation, the absorbance at 600 nm was measured, and the colony density was calculated.

## Live/dead bacterial staining assay

Live/dead bacterial staining assays were performed with different concentrations of CuMPBA after coincubation with bacteria in an acidic solution. The bacterial suspensions were stained with a live/dead bacterial activity kit containing N01 and PI (red and green fluorescence). Then, the cells were incubated under light-free conditions for 25 min. The dead and live bacteria were visualized with a laser scanning microscope (China-CSIM-130).

## Preparation of SEM for bacterial samples

The *MRSA* and *Sp* suspensions were diluted to 1 × 10^9^ CFU/mL. After treatment with and without hydrogen peroxide, the bacteria were incubated with different concentrations of CuMPBA in an acidic liquid medium. After centrifugation, the samples were washed three times with PBS. The supernatants of the centrifuged samples were subsequently discarded, and the remaining samples were fixed with 2.5% glutaraldehyde solution at 4 °C for 4 h. After complete fixation, the samples were centrifuged again. The supernatant was subsequently discarded, and the cells were washed with PBS 3 times. The samples were subsequently dehydrated in 30%, 50%, 70%, 90%, 95%, and 100% ethanol/water mixtures for 10 min. Finally, the freeze-dried samples were sputtered, gold-plated, and analyzed by scanning electron microscopy (Germany-ZEISS Sigma 360) and transmission electron microscopy (USA-FEI Talos F200X G2).

## Crystal violet staining

After the biofilms were treated via the previously described method, the medium was aspirated from the 96-well plate, and the plates were rinsed three times with PBS. The biofilm remaining at the bottom of the plate was subsequently fixed with 4% paraformaldehyde and further stained with 10% crystal violet solution for 20 min. Afterward, the staining solution was aspirated, the plates were washed to remove the floating color, and the stained biofilm was photographed with a camera for observation and subsequent solubilization with 30% ethanol. The absorbance was determined by using an enzyme marker to evaluate the biomass of the biofilm.

## Three-dimensional reconstruction of biofilms via CLSM

The biofilm was incubated in a confocal dish, and the three-dimensional structure of the biofilm was observed. After various treatments, they were washed and stained in a dark environment using the bestbio LIVE/DEAD Bacterial Activity Kit. 3D reconstruction was subsequently performed with a laser confocal microscopy imaging system (China-CSIM-130) and subsequently processed via ImageJ software.

## Hemolysis assessment

Red blood cells (RBCs) were collected from BALB/c mice and diluted to 2% with PBS. Different concentrations of the CuMPBA solutions were mixed with the erythrocyte suspensions and incubated on a shaker at 37 °C for 5 h. Then, the solutions were centrifuged at 2000 rpm for 10 min. Positive and negative controls were processed similarly for the diluted blood and were mixed directly with ultrapure water and saline. The absorbance of the supernatant at 540 nm was measured via an enzyme marker, and the hemolysis rate was calculated via the following formula:

Hemolysis rate (%) = (A_sample_ - A_neg_)/(A_pos_-A_neg_)*100

where A_sample_ is the absorbance of the different concentrations of CuMPBA, A_neg_ is the absorbance of the negative control, and A_pos_ is the absorbance of the positive control.

## *In vivo* ALI mouse model

An animal experiment with CuMPBA aimed to investigate its preventive effects on burn-induced ALI accompanied by pulmonary infection by constructing three different ALI models. The bacterium selected for the study was *Sp*, a common pulmonary resident bacterium. The animals were divided into the following groups: the control group, the LPS group, the LPS + bacteria + Burn group, and three treatment groups: the LPS + CuMPBA group, the LPS + bacteria + CuMPBA group, and the LPS + bacteria + Burn + CuMPBA group. Experimental procedure: a) Animal preparation: Female BALB/c mice aged 6–8 weeks were anesthetized with isoflurane and then fixed on a small animal operating table. Using a small animal laryngoscope, the airway was exposed, and the nebulizer needle was gently inserted into the airway to administer the drug. b) Treatment Administration: For the three treatment groups, a PBS solution containing CuMPBA was administered via airway nebulization at -2 hours before modeling. At 0 hours, the remaining seven groups were administered aerosolized drugs through the airway, with a total volume of 50 μL, consisting of 5 mg/kg LPS and 8.5 × 10^6^ CFU of bacteria. A 1 cm diameter circle of third-degree burn was induced on the mouse's back (85 °C, 8 seconds) according to the group allocation. c) Modeling and evaluation: On days 2 and 7, the remaining mice were sacrificed for pathological analysis to evaluate disease progression. On Day 2, the serum and bronchoalveolar lavage fluid (BALF) inflammatory cytokine levels were measured. All animal experiments were reviewed and approved by the Animal Care and Use Committee of Anhui Medical University (No. LLSC20242242).

## *In vivo* histologic analysis of an implant infection model

The tissues around the different groups of lungs were harvested at D2 and D7. The sections were subsequently analyzed via a Leica SP1600 microcomputer (Leica, Hamburg, Germany). Hematoxylin and eosin (H&E) were used to evaluate inflammation and bacterial residue. Immunohistochemical (IHC) staining was used to evaluate tissue inflammation. All the slides were observed under a light microscope (Olympus, IX70, Japan).

## *In vivo* biosafety assessment

Histologic observations of major organs (heart, liver, spleen, and kidneys) in different groups were performed via H&E staining and blood tests to evaluate the biosafety of these treatments.

## RNA-seq library preparation, sequencing, and data processing

The *Sp* was divided into two groups, each with three replicates, based on treatment with PBS or CuMPBA. The treated samples were subsequently sent to the Diogenes for detection and analysis. RNA was extracted from each treatment group via TRIzol reagent, with a total of 3 replicates per treatment. The samples were analyzed via an Illumina instrument. Fastq-formatted raw data (raw reads) were processed via an in-house Perl script. The reference genome and gene model annotation files were downloaded directly from the Genome website. The reference genome was indexed via Bowtie2-2.2.3, and the clean reads were compared with the reference genome. The number of reads corresponding to each gene was calculated via HTSeq v0.6.1. Differential expression analysis was performed for two conditions/groups (three biological replicates per condition) via the DESeq R package (1.18.0). Genes with a corrected P value < 0.05 were considered differentially expressed. Gene Ontology (GO) enrichment analysis of the differentially expressed genes was performed via the GOseq R package. GO terms with corrected P values < 0.05 were considered to be significantly enriched in DEGs. The GSEA tool (http://www.broadinstitute.org/gsea/index.jsp), the local version of GO, and the Kyoto Encyclopedia of Genes and Genomes (KEGG) dataset were used independently for GSEA.

## Statistical analysis

The data are expressed as the mean ± standard deviation (SD) for n ≥ 6 animals. Data were analyzed by one-way ANOVA followed by Tukey’s post hoc test, after confirming normality and homogeneity of variance and Tukey’s test. Differences between groups were considered to be statistically significant at *P<0.05, **P<0.01, and ***P<0.001. ns, not significant (P > 0.05).

**2. Supplementary figures**


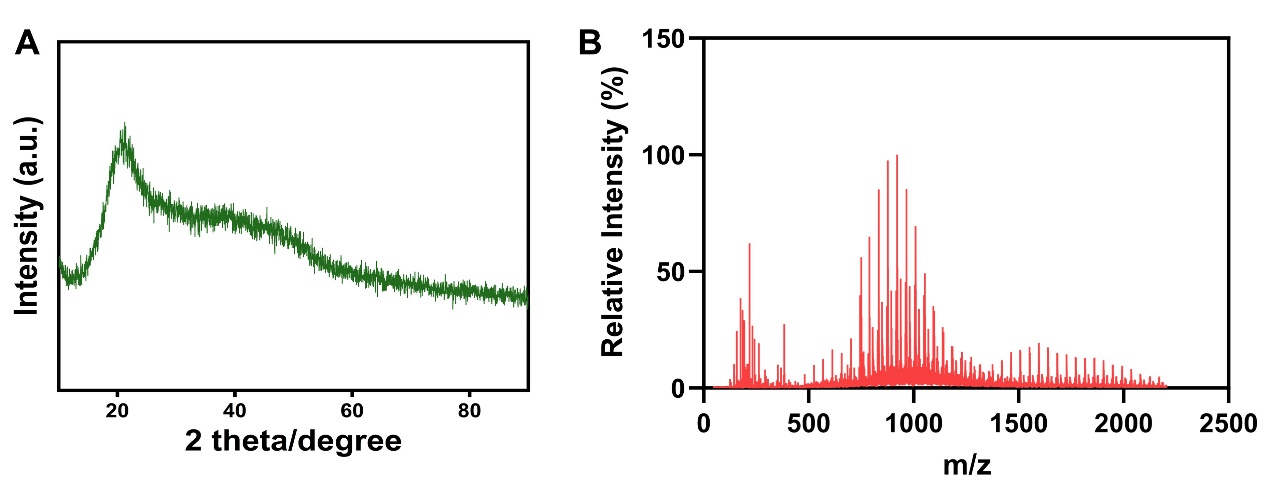


**Figure S1. Characterization of CuMPBA. (A)** XRD analysis of CuMPBA. **(B)** High-resolution mass spectrum of CuMPBA.


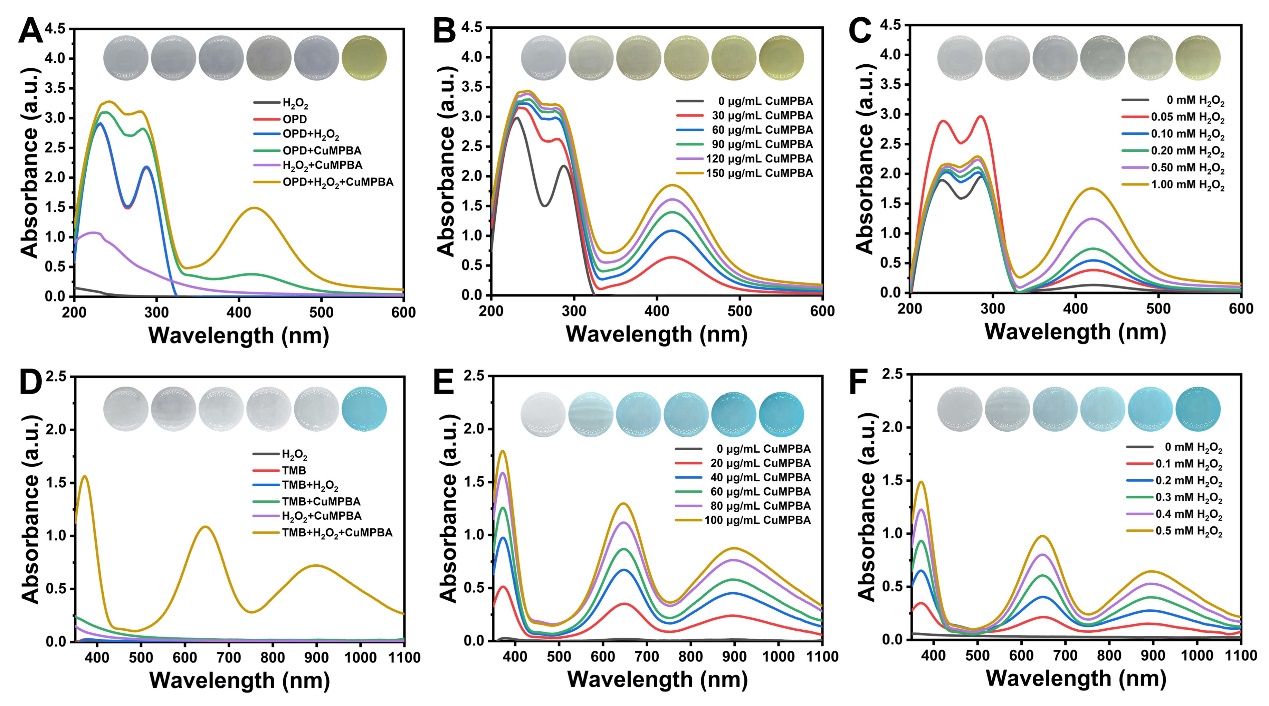


**Figure S2. Multienzyme activity of CuMPBA.** **(A)** UV‒vis spectra of solutions including OPD alone, H_2_O_2_, OPD, OPD+H_2_O_2_, CuMPBA+OPD, and CuMPBA+OPD+ H_2_O_2_ after 30 min of reaction. **(B)** POD-like activity; **(C)** POD-like activity of the CuMPBA nanoclusters using an OPD probe. **(D)** UV‒vis spectra of solutions including TMB alone, H_2_O_2_, TMB, TMB+ H_2_O_2_, CuMPBA + TMB, and CuMPBA+TMB+H_2_O_2_ after 30 min of reaction. **(E)** POD-like activity; **(F)** POD-like activity of CuMPBA nanoclusters using a TMB probe.


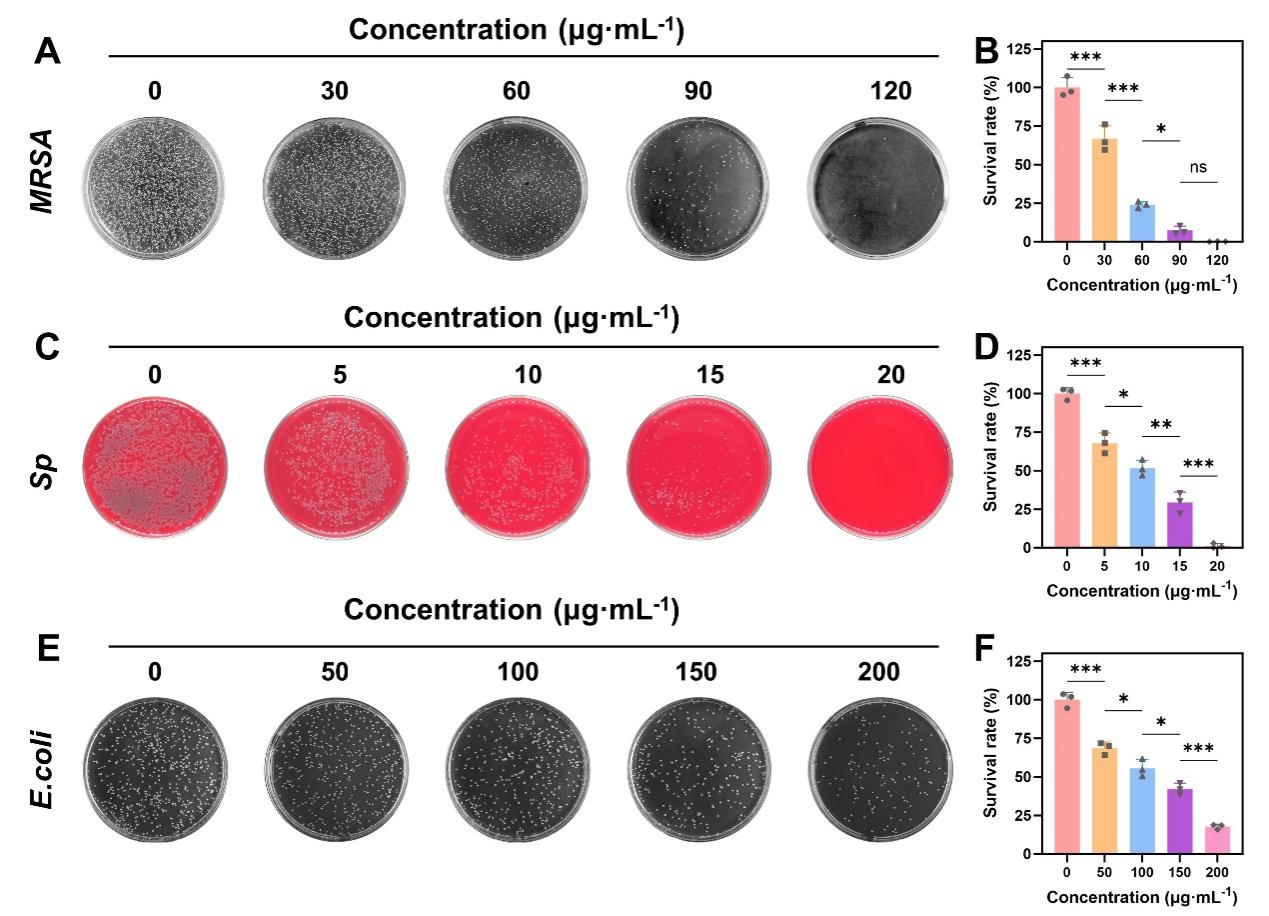


**Figure S3. Antibacterial performance of CuMPBA *in vitro*.** Photographs of bacterial colonies of *MRSA* **(A)***, Sp* **(C)** and *E. coli* **(E)**. **(B, D, F)** Statistical analysis of the results from (A, C, E). Data are presented as mean ± SD (n=3). *P < 0.05, **P < 0.01, ***P < 0.001; ns, not significant (P > 0.05).


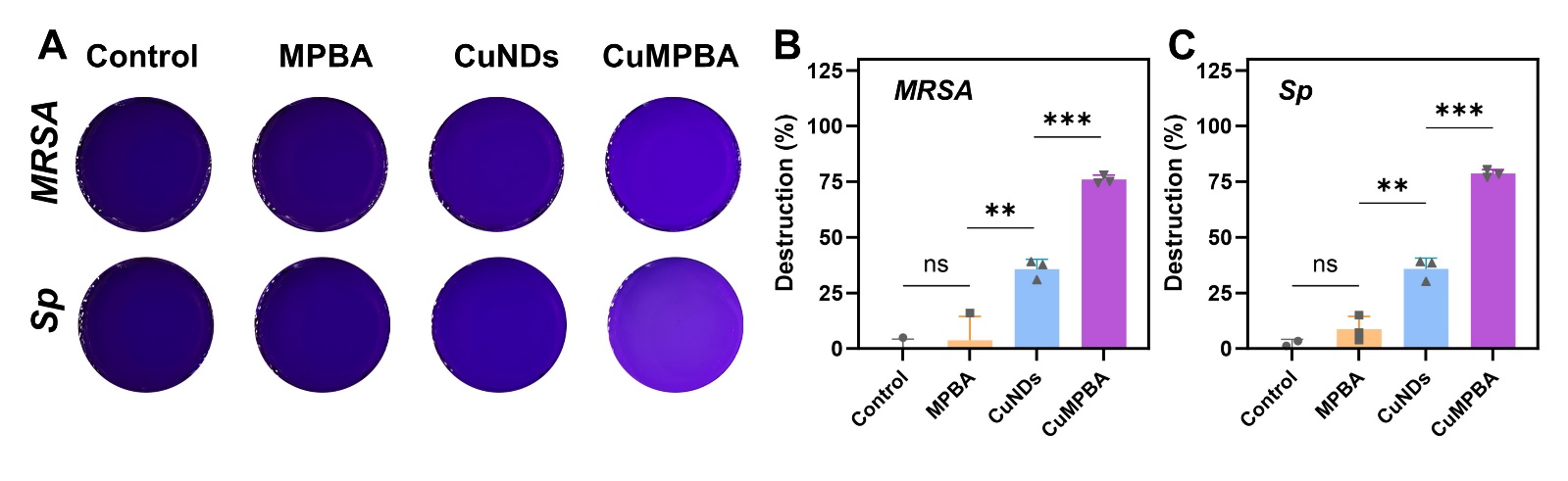


**Figure S4. *In vitro* evaluation of antibiofilm destruction efficiency. (A)** Verification of the effects of different components of CuMPBA on *MRSA* and *Sp* biofilms via crystal violet staining. **(B-C)** Statistical analysis of the results from (A). Data are presented as mean ± SD (n=3). *P < 0.05, **P < 0.01, ***P < 0.001; ns, not significant (P > 0.05).


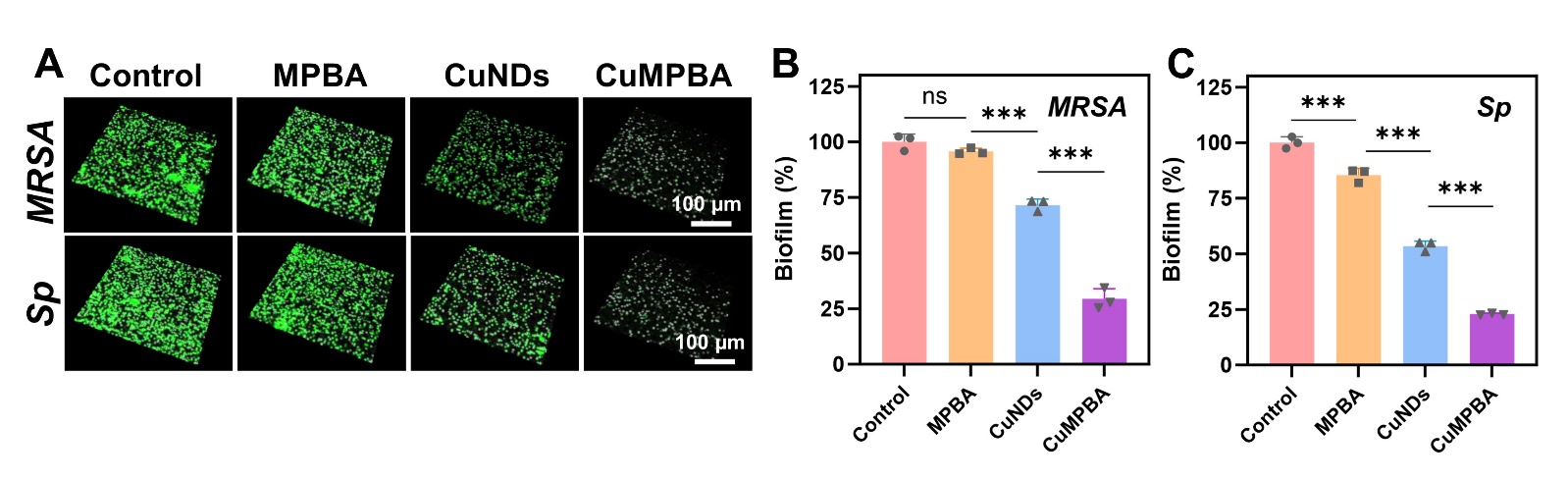


**Figure S5. *In vitro* evaluation of antibiofilm destruction efficiency. (A)** Fluorescence staining images and 3D reconstruction of the biofilm destruction experiment for *MRSA* and *Sp*. **(B-C)** Statistical analysis of the results from (A). Data are presented as mean ± SD (n=3). *P < 0.05, **P < 0.01, ***P < 0.001; ns, not significant (P > 0.05).


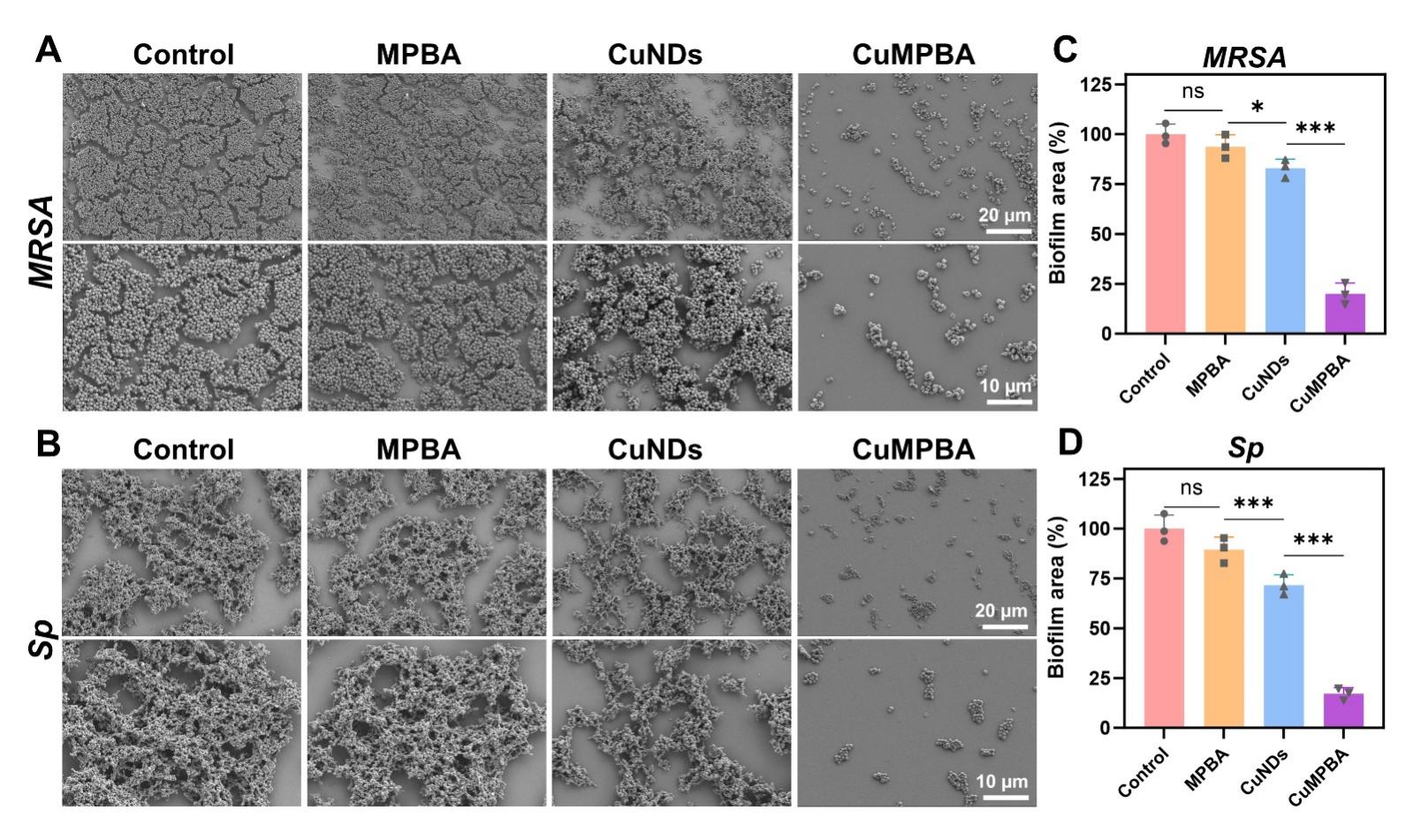


**Figure S6. *In vitro* evaluation of antibiofilm destruction efficiency. (A-B)** SEM analysis of the biofilm destruction of different components of CuMPBA against *MRSA* and *Sp*. **(C-D)** Statistical analysis of the results from (A, B). Data are presented as mean ± SD (n=3). *P < 0.05, **P < 0.01, ***P < 0.001; ns, not significant (P > 0.05).


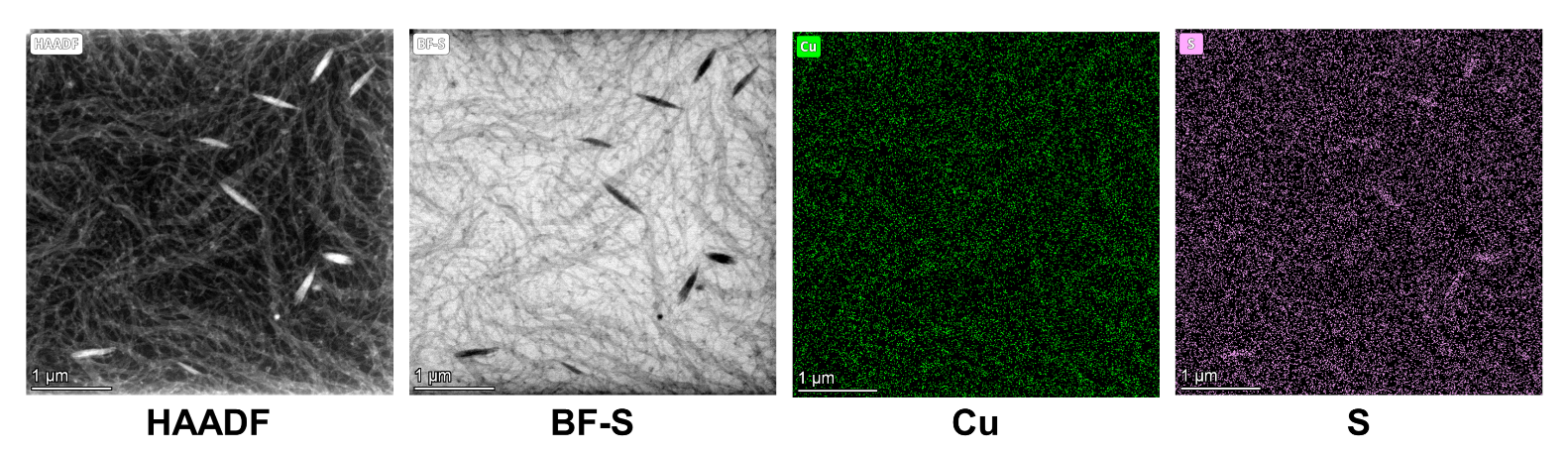


**Figure S7. Microstructure of CuMPBA reacted with LPS/LTA.** The TEM-EDS mapping results, through the enrichment of Cu and S elements, clearly indicate that the transformed fibrous structure originates from the originally spherical CuMPBA.


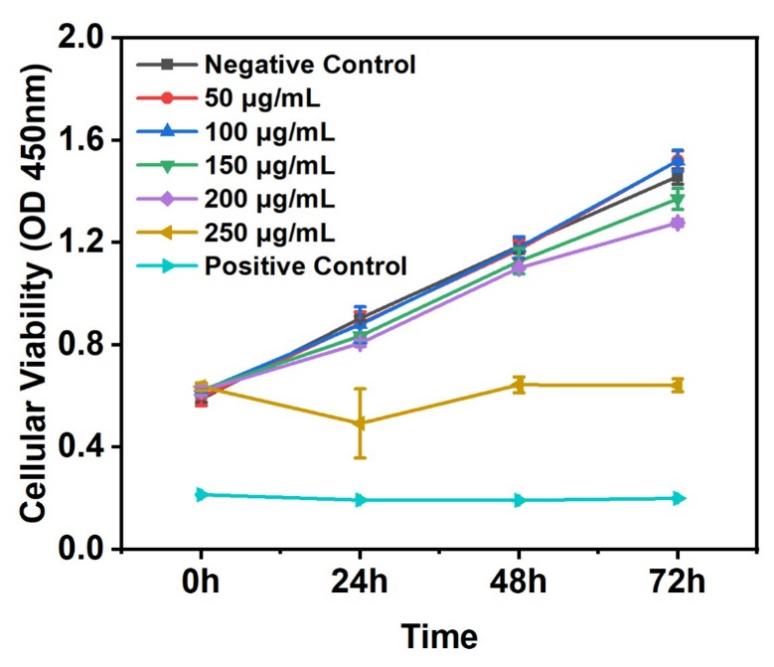


**Figure S8. Evaluation of *in vitro* cytotoxic activity.** CCK-8 assay of A549 lung epithelial cells treated with CuMPBA. Data are presented as mean ± SD (n=3).


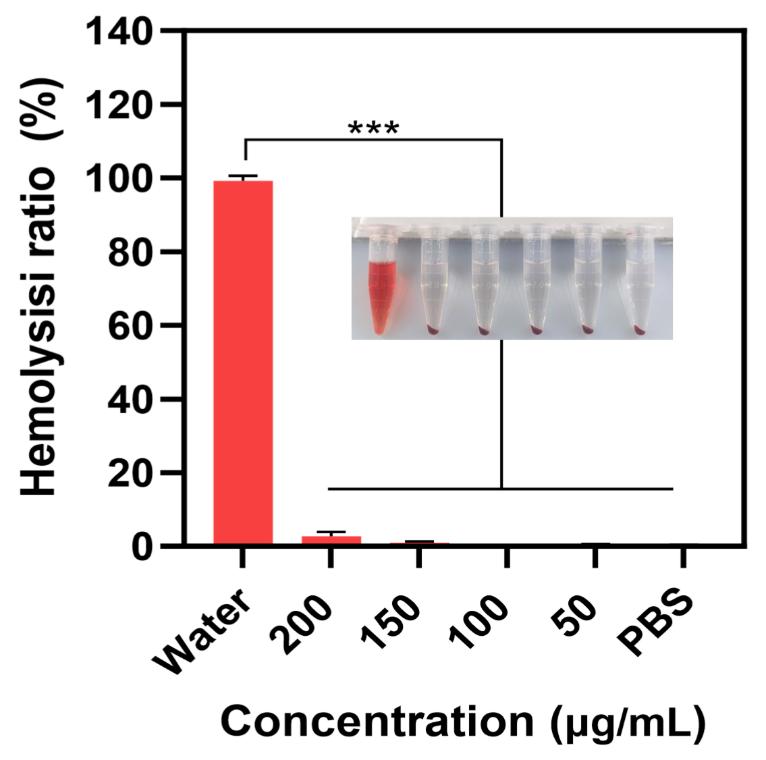


**Figure S9. Evaluation of *in vitro* cytotoxic activity.** Hemolysis experiment to verify the biosafety of CuMPBA. Data are presented as mean ± SD (n=3). *P < 0.05, **P < 0.01, ***P < 0.001; ns, not significant (P > 0.05).

**
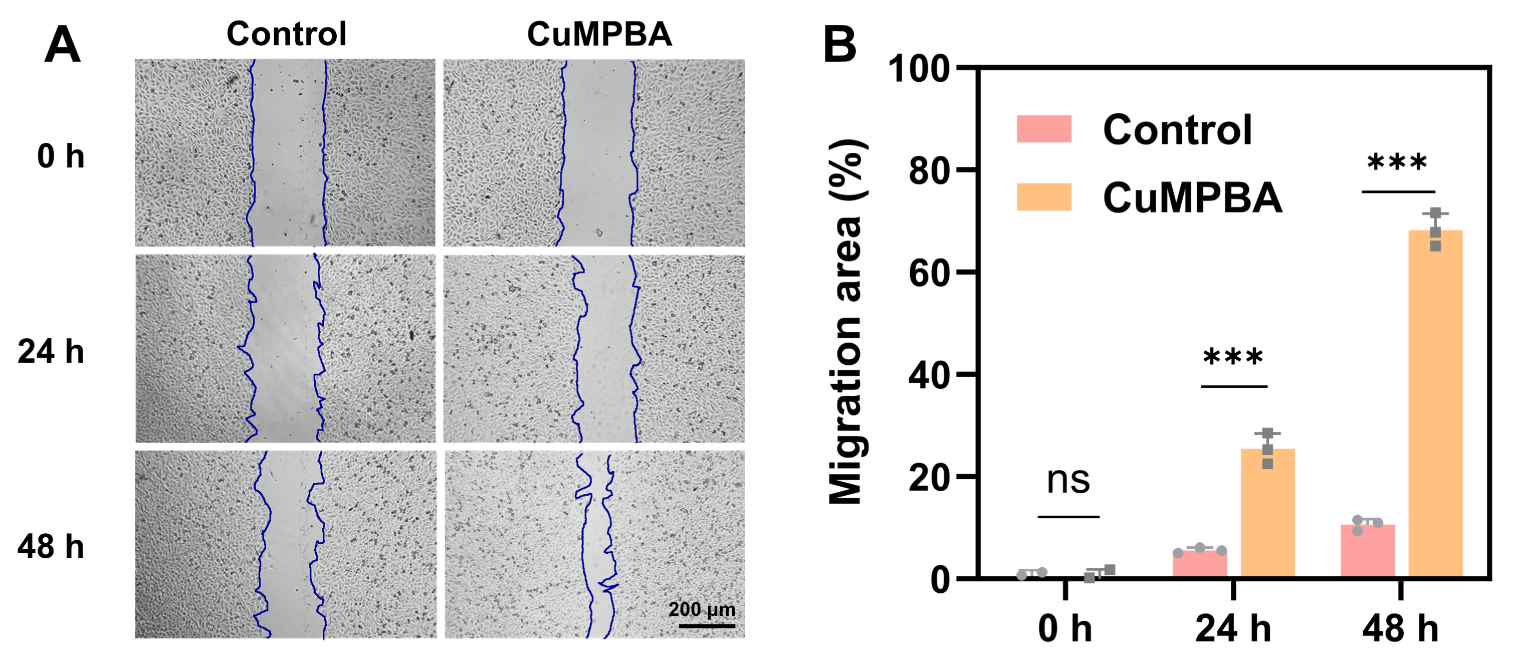
**

**Figure S10. Evaluation of** **the promotion of cell migration. (A)** The scratch assay was performed to test the ability of CuMPBA to promote the migration of HUVECs. **(B)** Statistical analysis of the migration area percentages from (A). Data are presented as mean ± SD (n=3). *P < 0.05, **P < 0.01, ***P < 0.001; ns, not significant (P > 0.05).


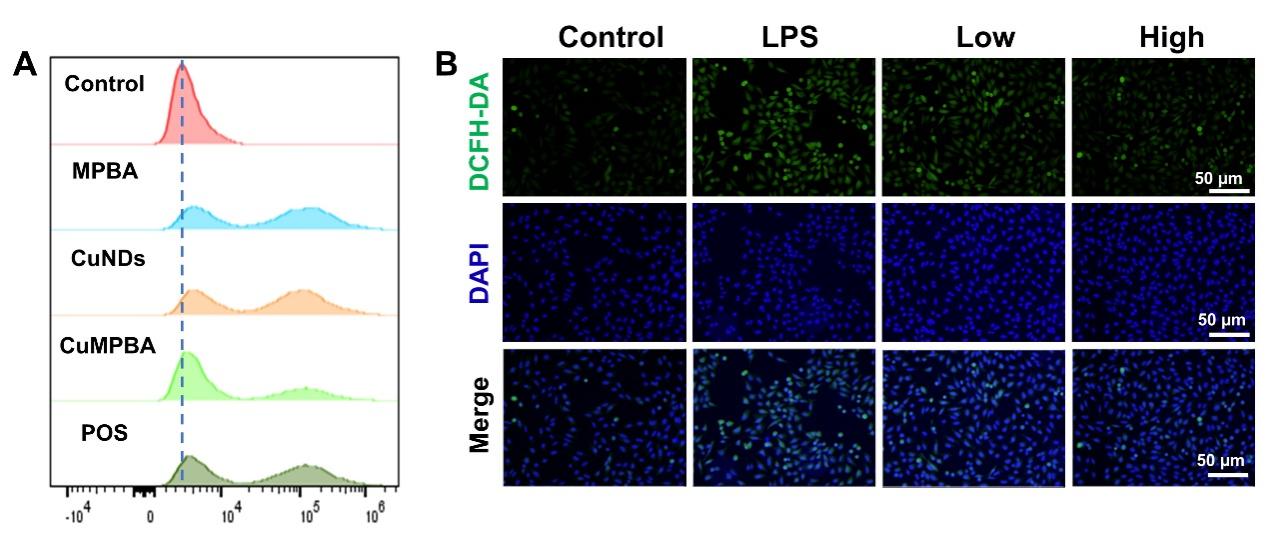


**Figure S11. Evaluation of anti-inflammatory activity *in vitro*. (A)** ROS levels were detected via flow cytometry to verify the protective effect of CuMPBA on A549 cells. **(B)** ROS levels were detected via fluorescence staining to verify the protective effect of CuMPBA on A549 cells.


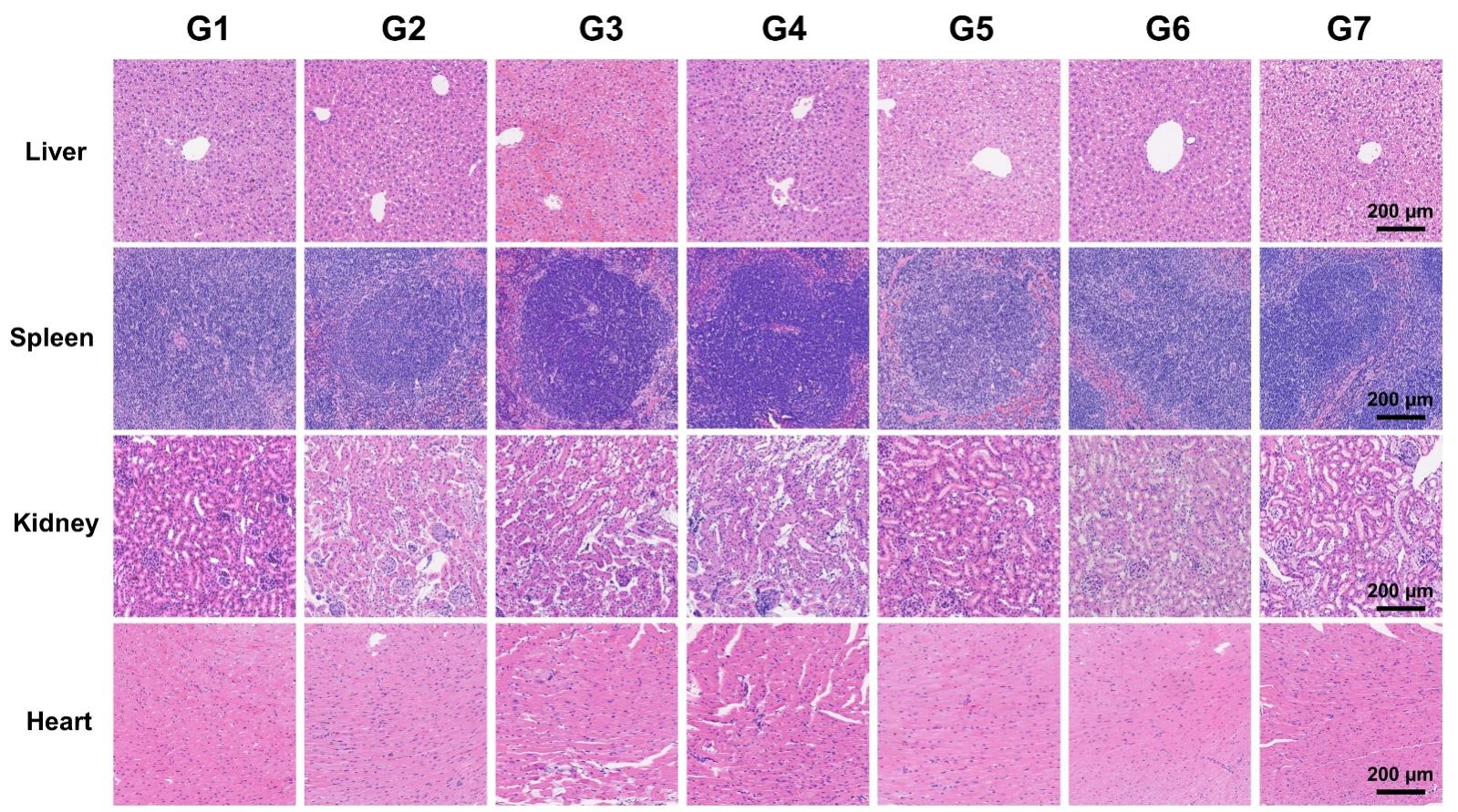


**Figure S12. H&E-stained sections of other organs (liver, spleen, kidney and heart) from the mice.** (Groups: G1: Control; G2: LPS; G3: LPS+*Sp*; G4: LPS+*Sp*+Burn; G5: LPS+CuMPBA; G6: LPS+*Sp*+CuMPBA; G7: LPS+*Sp*+Burn+CuMPBA)


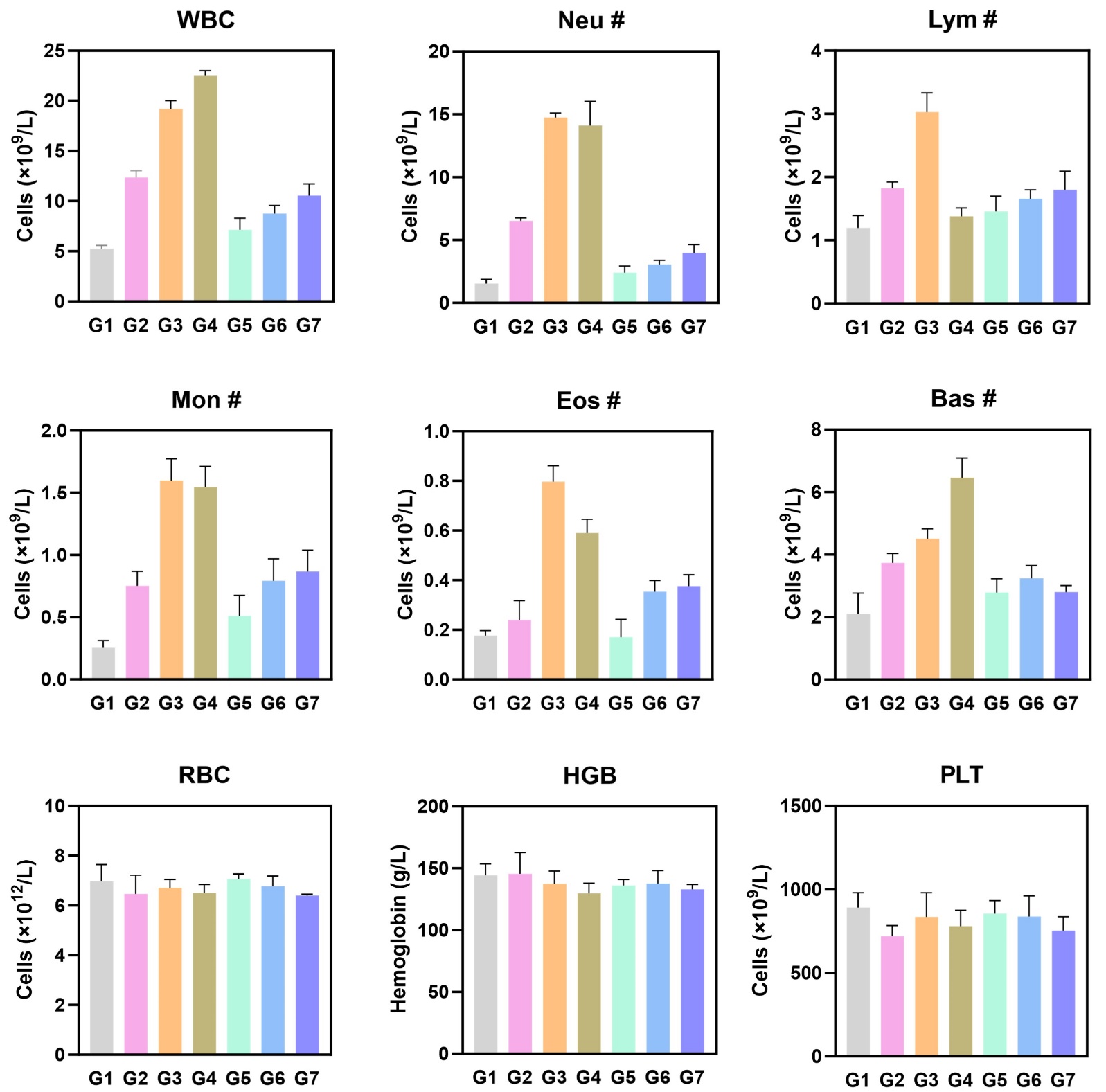


**Figure S13. Blood routine examination of mice.** Data are presented as mean ± SD (n=5). *P < 0.05, **P < 0.01, ***P < 0.001; ns, not significant (P > 0.05). (Groups: G1: Control; G2: LPS; G3: LPS+*Sp*; G4: LPS+*Sp*+Burn; G5: LPS+CuMPBA; G6: LPS+*Sp*+CuMPBA; G7: LPS+*Sp*+Burn+CuMPBA)


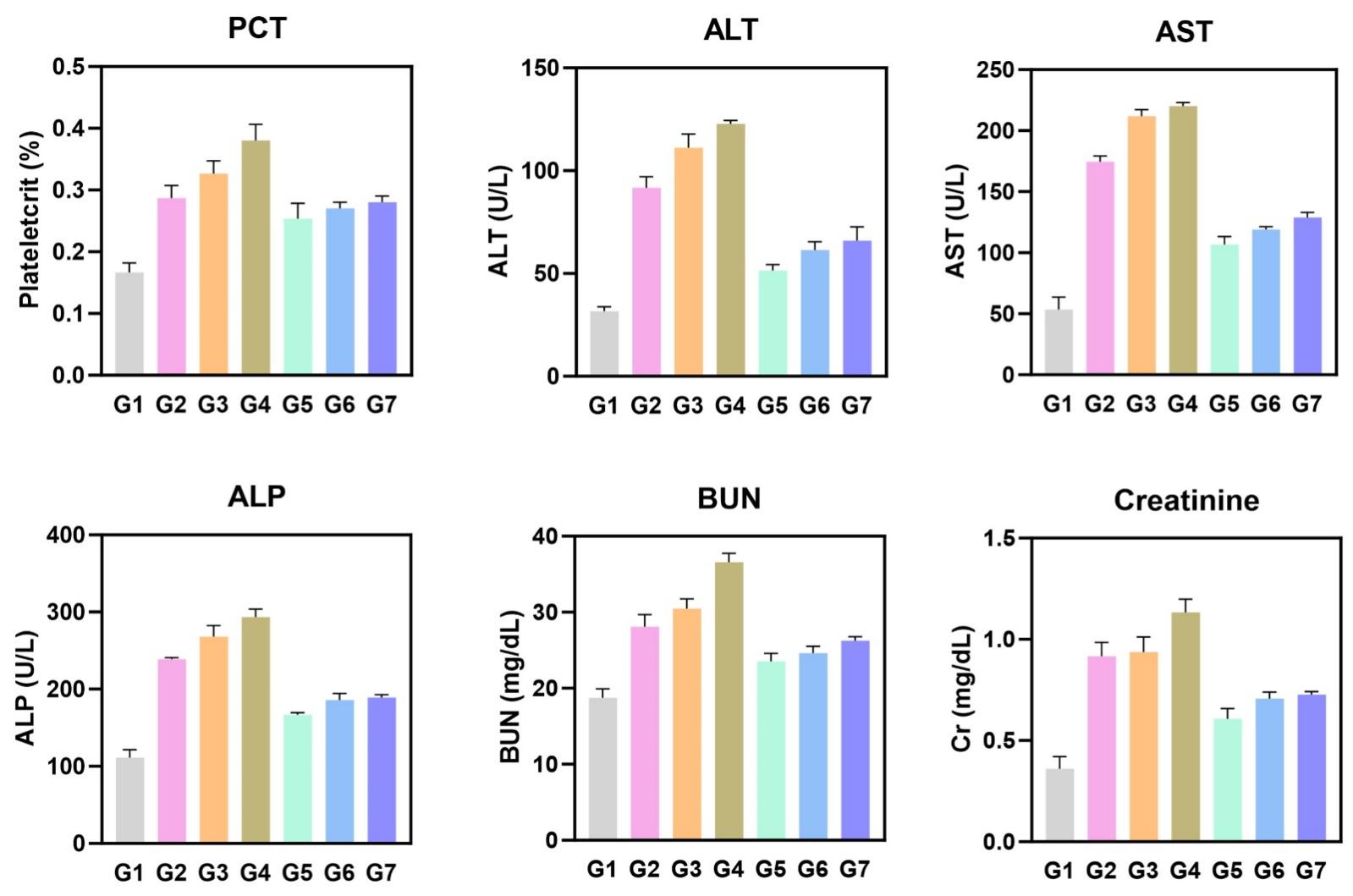


**Figure S14. Examination of procalcitonin and liver/kidney function in mice.** Data are presented as mean ± SD (n=5). *P < 0.05, **P < 0.01, ***P < 0.001; ns, not significant (P > 0.05). (Groups: G1: Control; G2: LPS; G3: LPS+*Sp*; G4: LPS+*Sp*+Burn; G5: LPS+CuMPBA; G6: LPS+*Sp*+CuMPBA; G7: LPS+*Sp*+Burn+CuMPBA)


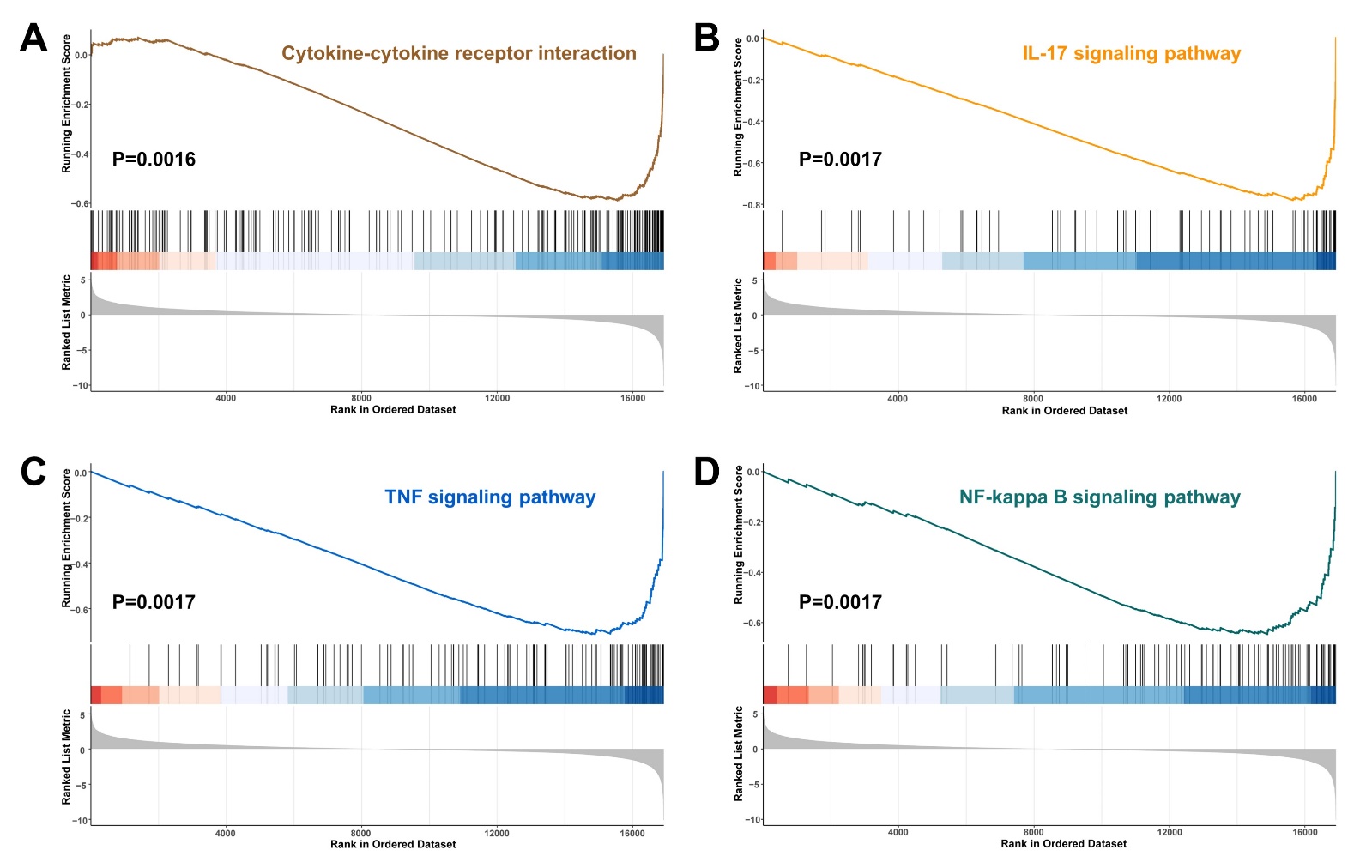


**Figure S15. The results of the GSEA enrichment analysis illustrated the enrichment of relevant inflammatory signaling pathways: (A)** Cytokine-cytokine receptor interaction pathway; **(B)** IL-17 signaling pathway; **(C)** TNF signaling pathway; **(D)** NF-κB signaling pathway. The y-axis represents the enrichment score, and the x-axis represents the rank of genes in the ordered dataset. The p-values in the figure are all less than 0.01, indicating significant negative enrichment of these pathways, suggesting that the expression of related genes is generally suppressed.
